# Tetrahydroxy‐Perylene Bisimide Embedded in a Zinc Oxide Thin Film as an Electron‐Transporting Layer for High‐Performance Non‐Fullerene Organic Solar Cells

**DOI:** 10.1002/anie.201907467

**Published:** 2019-08-14

**Authors:** Xinbo Wen, Agnieszka Nowak‐Król, Oliver Nagler, Felix Kraus, Na Zhu, Nan Zheng, Matthias Müller, David Schmidt, Zengqi Xie, Frank Würthner

**Affiliations:** ^1^ Institute of Polymer Optoelectronic Materials and Devices & State Key Laboratory of Luminescent Materials and Devices South China University of Technology Guangzhou 510640 P. R. China; ^2^ Institut für Organische Chemie & Center for Nanosystems Chemistry Universität Würzburg Am Hubland 97074 Würzburg Germany

**Keywords:** hydroxylation, metal complexes, perylene bisimide, photoconductive interlayer, solar cells

## Abstract

By introduction of four hydroxy (HO) groups into the two perylene bisimide (PBI) bay areas, new **HO‐PBI** ligands were obtained which upon deprotonation can complex Zn^II^ ions and photosensitize semiconductive zinc oxide thin films. Such coordination is beneficial for dispersing PBI photosensitizer molecules evenly into metal oxide films to fabricate organic–inorganic hybrid interlayers for organic solar cells. Supported by the photoconductive effect of the ZnO:**HO‐PBI** hybrid interlayers, improved electron collection and transportation is achieved in fullerene and non‐fullerene polymer solar cell devices, leading to remarkable power conversion efficiencies of up to 15.95 % for a non‐fullerene based organic solar cell.

During the last decade a variety of new materials have emerged that are promising candidates for solar energy conversion into electricity (photovoltaics) on a technological scale.^1^, ^2^, ^3^ In general, these photovoltaic devices rely on multiple layers which can either be inorganic or organic ones. However, quite often the organic/inorganic interface imposes some challenges. For instance, the widely applied zinc oxide (ZnO) wide band gap semiconductor interlayer in inverted bulk heterojunction polymer solar cells shows only modest conductivity which imposes a problem for the desired interlayer thicknesses of >100 nm. To solve this problem, recent approaches include doping with other metal ions^4^ and doping with photosensitizer dyes to afford a photoconducting interlayer.^5^

Inspired by the widely applied 1,1′‐bi‐2‐naphthol (BINOL)‐ligands for transition‐metal‐catalyzed asymmetric catalysis^6^ and an intriguing crystal structure published by Shibasaki and co‐workers for a trinuclear Zn^II^‐BINOL complex applied in catalytic asymmetric Mannich‐type reactions,^7^ we envisioned perylene bisimide (PBIs) dyes equipped with hydroxy functional groups in bay positions as coordinating ligands for Zn^2+^ ions for the incorporation of PBI dyes into ZnO semiconductor films^8^ manufactured by the sol‐gel process. However, whilst some twofold bay area hydroxylated PBIs have been reported,^9^ the desired tetrahydroxy PBIs do not yet exist. Herein, we report on new tetrahydroxy‐functionalized PBI dyes and their successful incorporation into ZnO interlayers to afford polymer solar cells with up to 15.95 % power conversion efficiency.

The synthesis of perylene bisimide dyes with suitable dihydroxylated ligation sites in both bay areas is shown in Scheme 1. This approach is based on our previously introduced copper‐catalyzed substitution of 1,6,7,12‐tetrabromo‐perylene dicarboxylic acid derivatives with sodium methoxide which is at best performed through the better soluble tetracarboxylic esters employing CuBr‐mediated cross‐coupling.^10^, ^11^ Subsequent conversion into **MeO‐PBI**s and ether cleavage with boron tribromide leads to the desired 1,6,7,12‐tetrahydroxy **HO‐PBI**s in yields of >80 %.

**Scheme 1:**
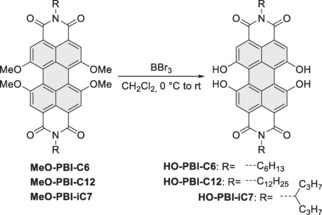
Synthesis of tetrahydroxy‐functionalized perylene bisimides.

In contrast to the red‐colored **MeO‐PBI**s, **HO‐PBI**s were isolated in color shades ranging from purple up to navy blue which is obviously a consequence of their high acidity, leading to easy deprotonation already in solvents such as methanol (for further details, see Supporting Information). Accordingly, the halochromic property^12^ of **HO‐PBI‐C12** was investigated in more detail by titration of a chloroform solution with 1,8‐diazabicyclo[5.4.0]undec‐7‐ene (DBU). As shown in Figure 1 fully protonated **HO‐PBI‐C12** in chloroform has a pink color and an absorption maximum at *λ*_max_=556 nm. Upon addition of DBU two subsequent deprotonation steps are observed, leading to a cyan‐colored solution at the end. As indicated by the presence of quasi‐isosbestic points for the spectral changes between 0 and 1 equivalent and between 1.5 and 5 equivalents of DBU, the first deprotonation is more favored, leading initially to a single deprotonated species (blue line, *λ*_max_=618 nm) that is further converted to a twofold deprotonated species (cyan line, *λ*_max_=640 nm). Therefore, it is most likely that each bay area is deprotonated only once whilst the second hydroxy group is not deprotonated by DBU base in chloroform.

**Figure 1:**
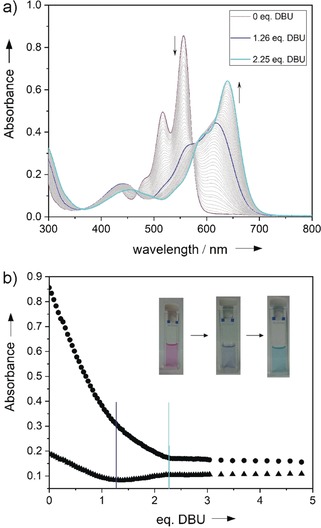
a) UV/Vis titration of **HO‐PBI‐C12** (*c*=1.9×10^−5^ m) in chloroform with DBU. b) Absorption changes at 488 nm (triangles) and 556 nm (circles) relative to the added equivalents of DBU and pictures of the cuvettes taken at the beginning (left), after addition of 1.3 equiv. DBU (middle) and at the end (right).

Owing to the difficulties encountered in the crystallization of **HO‐PBI**s our so far accomplished insights into the structural properties of these compounds rely on their **MeO‐PBI** precursors.10b Figure 2 shows the structure of **MeO‐PBI‐C6** in the single crystal. The most important features here are the dihedral angles ∡(C_1_−C_12b_−C_12a_−C_12_) and ∡(C_6_−C_6a_−C_6b_−C_7_) defined by the positions of the four bay carbon atoms of the PBI scaffold which are 30.0(2) and 29.6(2)°, respectively. Accordingly, the rotational twist between the two naphthalene subunits is close to 30° which is quite similar as observed previously for PBIs bearing four *n*‐butylthio‐substituents in bay area in chelate complexes with Pd^2+^ metal ions.^13^ Therefore and by taking into consideration a quite shallow potential energy surface with regard to the distortion of the two naphthalene subunits in PBIs,^14^ we may anticipate that **HO‐PBI**s are suitable chelate ligands for first row transition metals such as Zn^2+^ (which is demonstrated by our titration study for **HO‐PBI‐C12** with zinc triflate (Supporting Information, Figure S17). We also like to note that the spatial arrangement of the two coordinating oxygens in PBI bay area has a high resemblance to BINOL ligands used for Zn^2+^ complexation^7^ and the coordination sphere found in the wurtzite lattice of ZnO.^8^

**Figure 2:**
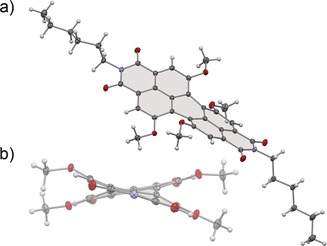
a) Solid‐state structure of **MeO**‐**PBI‐C6** determined by X‐ray analysis at 100 K. b) The view of the molecule along the long axis of the PBI core. ORTEP drawings are shown with 50 % probability. Imide substituents omitted for clarity. C grey, N blue, O red, H white.

The formation of ZnO interlayer films for inverted organic solar cells is typically carried out by spin‐coating method from a mixture of zinc acetate in 2‐methoxyethanol and 2‐aminoethanol, followed by thermal treatment.^15^ For solubility reasons, **HO‐PBI‐C12** and **HO‐PBI‐iC7** proved to be advantageous as they immediately dissolved upon their addition into this solvent mixture to give a pale blue color that is attributable to the deprotonation by the 2‐aminoethanol base. After spin‐coating of these precursor solutions and thermal treatment bluish colored thin films were obtained, whose absorption maxima (at 639 nm for **HO‐PBI‐C12**) are clearly different from those of pure **HO‐PBI** films but very similar to the twofold deprotonated species in our DBU titration (*λ*_max_=640 nm for **HO‐PBI‐C12**, see Figure 3).

**Figure 3:**
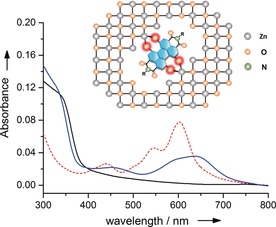
UV/Vis absorption spectra of **HO‐PBI‐C12** film (red dotted line), pristine ZnO (black line) and ZnO:**HO‐PBI‐C12** film (97:3 wt %, blue line) on quartz and schematic illustration of **HO‐PBI** dyes incorporated into the ZnO wurtzite lattice by O−Zn bonding.

The absorption spectra of the ZnO:**HO‐PBI** thin films accordingly suggests the incorporation of deprotonated **HO‐PBIs** within the ZnO semiconductor lattice (Figure 3). The pale blue color as well as the absorption spectra of the ZnO:**HO‐PBI** films keep unchanged even after being dipped into methanol, a good solvent for **HO‐PBI**s which demonstrates the strong and stable coordination between Zn^2+^ ions and PBI molecules through hydroxy groups. In the next step, inverted organic solar cells (OSCs) were fabricated with the device architecture of ITO/cathode interlayer/active layer/MoO_3_/Al, where the cathode interlayer indicates pure ZnO thin films or ZnO thin films doped with 1 wt % the new **HO‐PBI** molecules (Figure 4 b). We utilized fullerene‐based active layers (PTB7:PC71BM) as well as non‐fullerene‐based active layers (PBDB‐T‐2Cl:IT4F^16^ and PBDB‐T‐2F:Y6^17^) in the OSCs (Figure 4 a). Because the much better results were obtained for non‐fullerene acceptors our following discussion will focus on those whilst the data for the fullerene devices are collected in the Supporting Information. Table 1 summarizes the parameters of photovoltaic devices including open‐circuit voltage (*V*_oc_), short‐circuit current density (*J*_sc_), fill factor (*FF*), and power conversion efficiency (PCE). These parameters were obtained after optimization of doping ratio and annealing temperature of ZnO:PBI films (Supporting Information, Table S1, S2 and S3).

**Figure 4:**
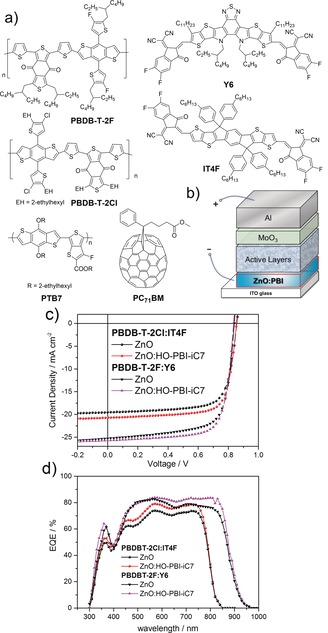
a) Donor polymers and acceptor molecules used in the active layers of inverted solar cells. b) Layer structure of the devices. c) *J*–*V* characteristics. d) EQE spectra of the devices based on PBDBT‐2Cl:IT4F and PBDB‐T‐2F:Y6 employing ZnO or ZnO:**HO‐PBI‐iC7** as the cathode interlayer.

**Table 1:** Photovoltaic parameters under AM1.5G simulated sunlight illumination of optimized solar cells employing different hybrid interlayers. The reported values were obtained from statistics of 10 devices.

Cathode interlayer	Active layers	*V*_oc_ [V]	*J_sc_* [mA/cm^2^]	*FF* [%]	PCE^[a]^ [%]
ZnO: **HO‐PBI‐C12**	PBDB‐T‐2Cl: IT4F	0.852	20.18	75.90	12.99 (13.17)
ZnO: **HO‐PBI‐iC7**	PBDB‐T‐2Cl: IT4F	0.860	20.41	75.58	13.26 (13.30)
ZnO: **HO‐PBI‐iC7**	PBDB‐T‐2F: Y6	0.830	25.34	74.80	15.73 (15.95)

[a] The best PCEs are provided in brackets.

The typical current density–voltage (*J*–*V*) curves for non‐fullerene active layer based devices are shown in Figure 4 c. The average PCEs of 12.99 % and 13.26 % was recorded for the PBDB‐T‐2Cl:IT4F based devices using ZnO:**HO‐PBI‐C12** and ZnO:**HO‐PBI‐iC7** as the cathode interlayers, respectively.

The slightly increased device performance for ZnO:**HO‐PBI‐iC7** devices might be attributed to the higher solubility and better molecular dispersion of these molecules in the ZnO thin films. It should be noted that a remarkable maximum PCE of 15.95 % was recorded when using PBDB‐T‐2F:Y6 as the active layer and ZnO:**HO‐PBI‐iC7** as the cathode interlayer, which is among the highest values reported in the literature. In contrast, devices employing pure sol‐gel‐derived ZnO as the cathode interlayer showed lower average PCEs of 12.04 % and 14.87 % (for detailed device parameters, see Table S4 in the Supporting Information) with PBDB‐T‐2Cl:IT4F and PBDB‐T‐2F:Y6, respectively. It is remarkable that the performance of solar cells with **HO‐PBI**‐embedded ZnO thin films showed a significant increase in *J*_sc_ and FF simultaneously, and that these interlayers work well for both fullerene (see Supporting Information) and non‐fullerene solar cells. These results demonstrate that the incorporation of hydroxylated PBI dyes into inorganic n‐type ZnO thin films affords an improvement of the cathode interlayer due to robust coordination between organic molecules and the metal oxide lattice, which leads to increased electron mobility and easier photo‐induced electron transfer from PBI molecule to ZnO for better electron transporting properties under light illumination (for further physical characterization, see the Supporting Information).^5^

Figure 4 d shows the external quantum efficiency (EQE) spectra of two different non‐fullerene solar cells. The solar cells have a broad spectral response and the maxima of EQEs are as high as about 80 % and 85 % for PBDB‐T‐2Cl:IT4F and PBDB‐T‐2F:Y6 based devices, respectively. The calculated *J*_sc_ integrated by EQE spectra were 19.60 mA cm^−2^ and 25.1 mA cm^−2^, which correspond well to the *J*–*V* characteristics of above devices. When compared with ZnO based devices, ZnO:**HO‐PBI‐iC7** device shows an obvious increase of EQE in most of the spectral region and does not show a loss in the PBI absorption region. In addition, the application of such hybrid interlayers can increase the rectification ratio of organic solar cells according to the dark *J*–*V* curve measurements, which might be attributed to the enhanced hole blocking ability of the hybrids (Supporting Information, Figure S21).

In summary, the coordinative properties of new bay‐functionalized tetrahydroxy‐perylene bisimides could be utilized for the manufacture of photoconductive ZnO thin films. Owing to the tight binding of the deprotonated PBI dyes very robust hybrid thin films could be obtained as required for the fabrication of multiple layered devices by solution processing. The tetrahydroxy‐PBI embedded ZnO thin films were applied as cathode interlayers in inverted non‐fullerene OSCs to give PCEs up to 15.95 %. These results show that the coordination between hydroxy‐PBI dyes and metal ions through hydroxyl functional groups offers an attractive approach towards high performance photoconductive electron transport interlayers. We envision that our concept might be also applicable to other low‐band gap semiconductors including titanium dioxide as well as for interlayers used in OLED devices.

## Conflict of interest

The authors declare no conflict of interest.

## Supporting information

As a service to our authors and readers, this journal provides supporting information supplied by the authors. Such materials are peer reviewed and may be re‐organized for online delivery, but are not copy‐edited or typeset. Technical support issues arising from supporting information (other than missing files) should be addressed to the authors.

Supplementary
