# Supplementary material for: Tetrahydroxy‐Perylene Bisimide Embedded in a Zinc Oxide Thin Film as an Electron‐Transporting Layer for High‐Performance Non‐Fullerene Organic Solar Cells
Source: Angew Chem Int Ed Engl. 2019 Aug 14;58(37):13051–5. doi: 10.1002/anie.201907467 (PMC6772159; doi:10.1002/anie.201907467)
Supplement: Supplementary file 1 — Supplementary [file ANIE-58-13051-s001.pdf]

Angewandte Chemie
Eine Zeitschrift der Gesellschaft Deutscher Chemiker

Supporting Information

Tetrahydroxy-Perylene Bisimide Embedded in Zinc Oxide Thin Film as Electron Transporting Layer for High Performance Non-Fullerene Organic Solar Cells

Xinbo Wen+, Agnieszka Nowak-Król+, Oliver Nagler, Felix Kraus, Na Zhu, Nan Zheng, Matthias Müller, David Schmidt, Zengqi Xie, and Frank Würthner**

anie_201907467_sm_miscellaneous_information.pdf

Table of Contents

1. Materials and Methods S3
2. Synthesis and Characterization of Tetrahydroxy-PBIs S3
3. Solvent and pH-dependent absorption spectra of HO-PBI-C12 .... S12
4. Coordination of Tetrahydroxy-PBIs and Zn(II) Metal ions S13
5. Electrochemistry S14
6. Single crystal X-ray analysis S15
7. Fabrication and Characterization of OSCs S15
   7.1 Fabrication and Characterization Method of Devices S15
   7.2 Photovoltaic Performance of OSCs with Various Cathode Interlayers and BHJ Layers S16
   7.3 Electron Transporting Properties of the Cathode Interlayers S18
   7.4 Surface Morphology of Cathode Interlayer Thin Films S20

References S20

1. Materials and Methods

General: All reagents were purchased from commercial sources and used as received without further purification, unless otherwise stated. 1,6,7,12-Tetramethoxyperylene-3,4:9,10-tetracarboxylic acid bisanhydride and **MeO-PBI-C12** were prepared according to the reported procedures.[S1,S2] Reagent grade solvents were distilled prior to use. Column chromatography was performed on silica (silica gel, 230–400 mesh). ^{1}H, ^{13}C NMR spectra were recorded on a Bruker Avance 400 or an Avance III HD 400 spectrometers and were calibrated to the residual solvent signals. *J* values are given in Hz. The following abbreviations were used to designate multiplicities: s = singlet, t = triplet, m = multiplet, br s = broad singlet. High resolution mass spectra were obtained by electrospray ionization (ESI) or matrix-assisted laser desorption/ionization (MALDI). ESI spectra were recorded on an ESI micrOTOF Focus spectrometer from Bruker Daltonics. MALDI spectra were recorded on a Bruker Daltonics autoflex II LRF or a Bruker Daltonics ultrafleXtreme spectrometers. *Trans*-2-[3-(4-tert-butylphenyl)-2-methyl-2-propenylidene]malononitrile (DCTB) or 2',4'-dihydroxyacetophenone (DHAP) were used as MALDI matrices. UV/Vis spectra were recorded on a Jasco V-770 UV/Vis spectrometer. All spectroscopy measurements were conducted with spectroscopic grade solvents from ACROS Organics. Conventional quartz cells (light path 1 cm) were used. pH was measured using a Mettler Toledo FiveGo pH meter FG2 equipped with a SI Analytics N 5800 BNC electrode. The CV measurements were performed on a standard, commercial electrochemical analyzer (EC epsilon; BAS Instruments, UK) in a three electrode single-compartment cell under an argon atmosphere. The supporting electrolyte NBu_4PF_6 was synthesized according to the literature,[S3] recrystallized from ethanol/water, and dried in a high vacuum. The measurements were carried out in methanol/0.1m NBu_4PF_6 under the exclusion of air and moisture at a concentration of $c \sim 2.8\cdot10^{-4}$ M with the ferrocenium/ferrocene redox couple as an internal standard for the calibration of the potential. Working electrode: Pt disc (Ø 1 mm); reference electrode: Ag/AgCl; auxiliary electrode: Pt wire. The internal resistance was compensated by 50%.

2. Synthesis and Characterization of Tetrahydroxy-PBIs

Synthesis of *N*,*N*'-di(heptan-4-yl)-1,6,7,12-tetramethoxyperylene-3,4:9,10-tetracarboxylic acid bisimide (MeO-PBI-iC7).

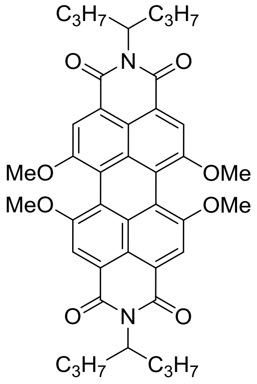

1,6,7,12-Tetramethoxyperylene-3,4:9,10-tetracarboxylic acid bisanhydride (34 mg, 0.07 mmol) was dissolved in molten imidazole (1 g) and 4-heptylamine (100 µL, 0.67 mmol) was added. The mixture was stirred for 24 h at 120 °C and then poured into 2M HCl (100 mL). The aqueous phase was extracted with CH_2Cl_2. The organic extracts were combined and washed with $NaHCO_3$ (aq.), dried over Na_2SO_4, filtered and evaporated. The crude product was purified by column chromatography (silica, CH_2Cl_2) to afford **MeO-PBI-iC7** (24 mg, 51 %) as a navy blue solid. M.p.: 395-396 °C, ^{1}H NMR (400 MHz, $CDCl_3$, 298 K): δ=8.32 (br s, 4H), 5.31-5.23 (m, 2H), 4.21 (s, 12H), 2.35-2.26 (m, 4H), 1.84-1.75 (m, 4H), 1.40-1.25 (m, 8H), 0.92 ppm (t, *J*=7.4 Hz, 12H); ^{13}C NMR (101 MHz, $CDCl_3$, 298 K): δ=165.4, 164.4, 156.8, 132.3, 122.8, 122.0, 119.0, 118.5, 115.1, 114.4, 56.8, 54.1, 34.7, 20.2, 14.2 ppm; HRMS (MALDI-TOF): m/z calcd for $C_{42}H_{46}N_2O_8$ $M^{\bullet+}$:706.3248; found: 706.3283.

Synthesis of *N*,*N*'-di(heptan-4-yl)-1,6,7,12-tetrahydroxyperylene-3,4:9,10-tetracarboxylic acid bisimide (HO-PBI-iC7).

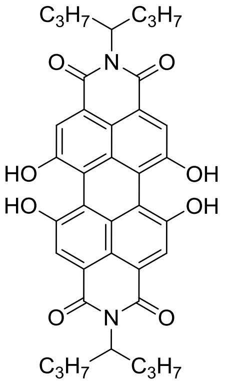

MeO-PBI-iC7 (30 mg, 0.04 mmol) was dissolved in anhydrous CH_2Cl_2 (7 mL) and BBr_3 (0.20 mL, 2.12 mmol) in anhydrous CH_2Cl_2 (3 mL) was added at 0 °C. After 1 h at 0 °C the mixture was stirred at room temperature for 5 d. Afterwards a solution of MeOH in water (1:4, 10 mL) was added, the precipitate was filtered and washed with water, CH_2Cl_2 and *n*-pentane. **HO-PBI-iC7** (23 mg, 83 %) was obtained as a dark violet solid. M.p.: >400 °C, ^{1}H NMR (400 MHz, $CDCl_3/CD_3OD$ 10:1, 298 K): δ=8.16 (br s, 4H), 5.20-5.13 (m, 2H), 2.25-2.15 (m, 4H), 1.79-1.70 (m, 4H), 1.35-1.14 (m, 8H), 0.86 ppm (t, *J*=7.3 Hz, 12H); ^{13}C NMR (101 MHz, $CDCl_3/CD_3OD$ 10:1, 298 K): δ=165.5, 164.4, 152.8, 132.2, 122.8, 122.1, 121.7, 121.0, 118.7, 118.4, 54.2, 34.5, 20.1, 13.9 ppm; HRMS (MALDI-TOF): m/z calcd for $C_{38}H_{38}N_2O_8$ $M^{\bullet+}$: 650.2623; found: 650.2716.

Synthesis of *N*,*N*'-didodecyl-1,6,7,12-tetrahydroxyperylene-3,4:9,10-tetracarboxylic acid bisimide (HO-PBI-C12).

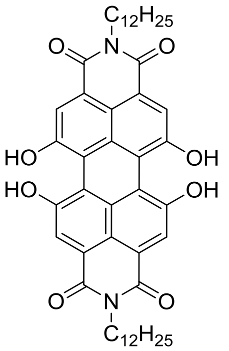

MeO-PBI-C12 (102 mg, 0.12 mmol) was dissolved in anhydrous CH_2Cl_2 (18 mL) and BBr_3 (0.57 mL, 6.01 mmol) in anhydrous CH_2Cl_2 (10 mL) was added at 0 °C. After 1 h at 0 °C the mixture was stirred at room temperature for 6 d. Afterwards a solution of MeOH in water (1:4, 20 mL) was added, the precipitate was filtered and washed with water, CH_2Cl_2 and *n*-pentane. Product **HO-PBI-C12** (81 mg, 85 %) was obtained as a dark violet solid. M.p.: >400 °C, ^1H NMR (400 MHz, $CDCl_3/CD_3OD$ 10:1, 298 K): δ=8.13 (s, 4H), 4.10 (t, *J*=7.6 Hz, 4H), 1.65 (q, *J*=7.3 Hz, 4H), 1.39-1.16 (m, 36H), 0.78 ppm (t, *J*=6.9 Hz, 6H); ^{13}C NMR (101 MHz, $CDCl_3/CD_3OD$ 10:1, 298 K): δ=164.1, 152.6, 132.0, 122.2, 121.1, 118.6, 118.1, 40.7, 31.9, 29.62, 29.59, 29.5, 29.4, 29.3, 28.1, 27.1, 22.6, 14.0 ppm; HRMS (MALDI-TOF): m/z calcd for $C_{48}H_{59}N_2O_8$ $[M + H]^+$: 791.4266; found: 791.4276. The isotopic pattern is the sum of overlapping isotopic patterns of $M^{•+}$, $[M + H]^+$ and $[M + D]^+$.

Synthesis of the sodium salt of HO-PBI-C12.

MeO-PBI-C12 (50 mg, 06 mmol) was dissolved in anhydrous CH_2Cl_2 (7.2 mL) under nitrogen and cooled to 0 °C. A solution of BBr_3 (0.25 mL, 2.63 mmol) in anhydrous CH_2Cl_2 (3.8 mL) was prepared in a separate vessel and cooled to 0 °C. Next, the ice-cold solution of BBr_3 was added dropwise to the solution of **MeO-PBI-C12** and the reaction mixture was stirred at 0 °C under nitrogen for 1 h. Then stirring was continued at room temperature for 32 h. Afterwards water and acetone were added and the precipitate was filtered. The solid was washed with water and CH_2Cl_2. Next the solid was washed with acetone, $NaHCO_3$ (aq.) and water, then CH_2Cl_2 and pentane. The residue was dissolved in MeOH and crystallized (MeOH/toluene/CH_2Cl_2) to give **HO-PBI-C12** (41 mg, 85 %) as a navy blue solid. M.p.: >400 °C; ^1H NMR (400 MHz, CD_3OD, 326 K): δ=8.12 (s, 4H), 4.20 (t, *J*=7.4 Hz, 4H), 1.92-1.64 (m, 4H), 1.54-1.17 (m, 36H), 0.97-0.81 ppm (m, 6H); ^{13}C NMR (101 MHz, CD_3OD, 326 K): δ=165.7, 159.0, 133.0, 122.4, 122.0, 120.2, 115.3, 39.9, 31.7, 29.4, 29.3, 29.3, 29.1, 29.0, 27.9, 26.9, 22.3, 13.0 ppm. HRMS (ESI-TOF): m/z calcd for $C_{48}H_{57}N_2O_8$ [**O-PBI-C12**]$^-$: 789.4109; found: 789.4126; LRMS (MALDI-TOF): m/z calcd for $C_{48}H_{58}N_2O_8$ [**HO-PBI-C12**]$^{•+}$: 790.42; found: 790.38.

Synthesis of *N*,*N*'-dihexyl-1,6,7,12-tetramethoxyperylene-3,4:9,10-tetracarboxylic acid bisimide (MeO-PBI-C6).

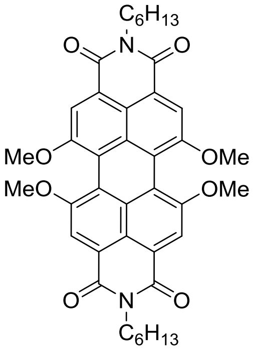

1,6,7,12-Tetramethoxyperylene-3,4:9,10-tetracarboxylic acid bisanhydride (40 mg, 0.078 mmol) was dissolved in propionic acid (4.0 mL) and *n*-hexylamine (215 µL, 1.64 mmol) was added. The reaction mixture was stirred at 120 °C for 28.5 h and then poured into water. Phases were separated. The aqueous phase was extracted with CH_2Cl_2. The organic extracts were combined and washed with $NaHCO_3$ (aq.), dried over Na_2SO_4, filtered and evaporated. The crude product was purified by column chromatography (silica, CH_2Cl_2) and crystallized (CH_2Cl_2, pentane) to afford **MeO-PBI-C6** (46 mg, 87 %) as wine red crystals. M.p.: 362-363 °C, ^1H NMR (400 MHz, $CDCl_3$, 298 K): δ=8.33 (s, 4H), 4.38-4.01 (m, 16H), 1.86-1.68 (m, 4H), 1.53-1.23 (m, 12H), 0.90 ppm (t, *J* = 7.1 Hz, 6H); ^{13}C NMR (101 MHz, $CDCl_3$, 298 K): δ=164.1, 156.9, 132.3, 122.2, 119.2, 114.6, 56.8, 40.8, 31.7, 28.3, 26.9, 22.7, 14.2 ppm; HRMS (ESI-TOF): m/z calcd for $C_{40}H_{42}N_2O_8$ $M^{•+}$: 678.2936; found: 678.2930.

Synthesis of *N*,*N*'-dihexyl-1,6,7,12-tetrahydroxyperylene-3,4:9,10-tetracarboxylic acid bisimide (HO-PBI-C6).

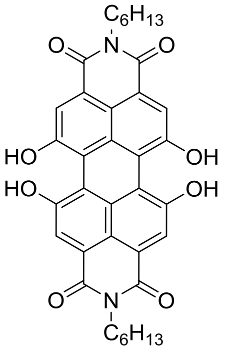

MeO-PBI-C6 (20 mg, 0.03 mmol) was dissolved in anhydrous CH_2Cl_2 (5 mL) and BBr_3 (0.13 mL, 1.47 mmol) in anhydrous CH_2Cl_2 (1 mL) was added at 0 °C. After 1 h at 0 °C the mixture was stirred at room temperature for 6 d. Afterwards a solution of MeOH in water (1:4, 5 mL) was added, the precipitate filtered and washed with water, CH_2Cl_2 and *n*-pentane. **HO-PBI-C6** (15 mg, 82 %) was obtained as a dark violet solid. M.p.: >400 °C, ^1H NMR (400 MHz, $CDCl_3/CD_3OD$ 10:1, 298 K): δ=8.09 (s, 4H), 4.25-3.95 (m, 4H), 1.81-1.55 (m, 4H), 1.48-1.06 (m, 12H), 0.94-0.62 ppm (m, 6H); ^{13}C NMR not measured due to low solubility; HRMS (MALDI-TOF): m/z calcd for $C_{36}H_{33}N_2O_8$ $[M - H]^-$: 621.2242; found: 621.2604.

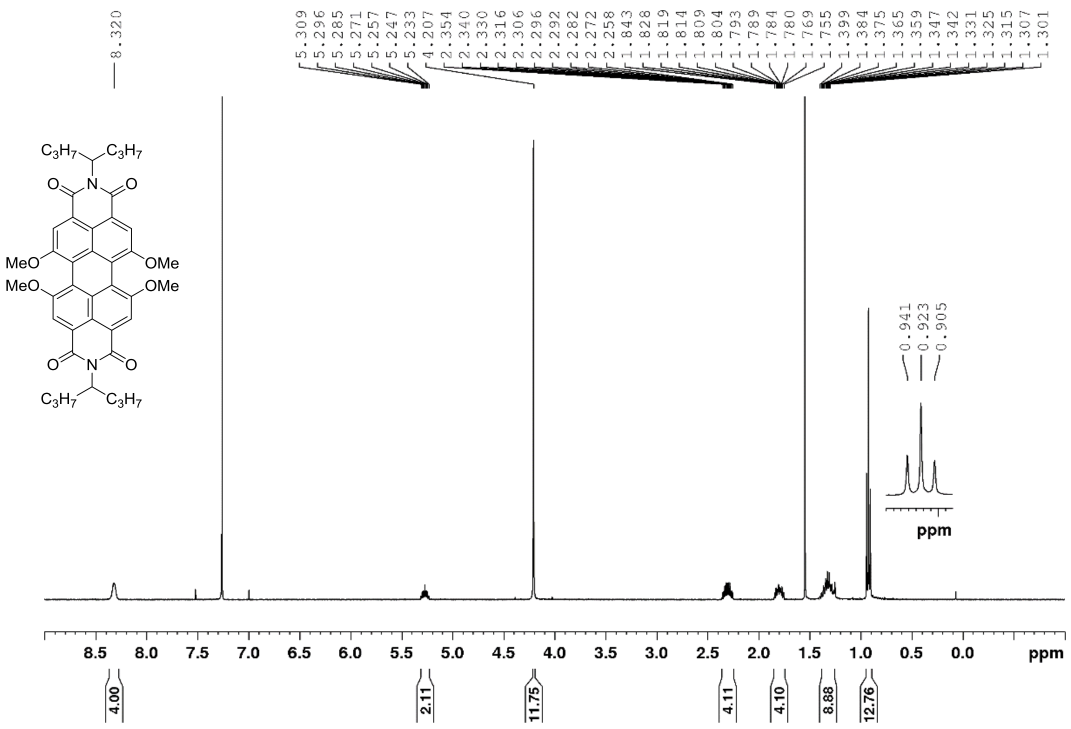

Figure S1. ^1H NMR of compound **MeO-PBI-iC7** (400 MHz, $CDCl_3$, 298 K).

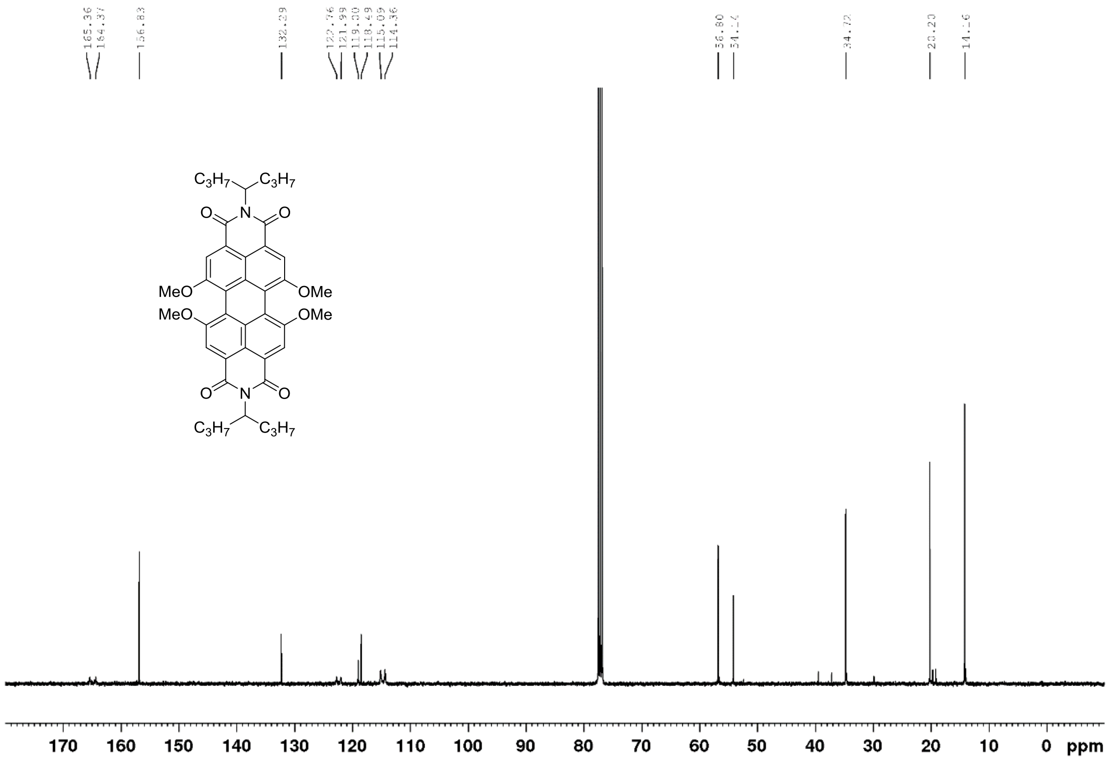

Figure S2. ^{13}C NMR of compound **MeO-PBI-iC7** (101 MHz, $CDCl_3$, 298 K).

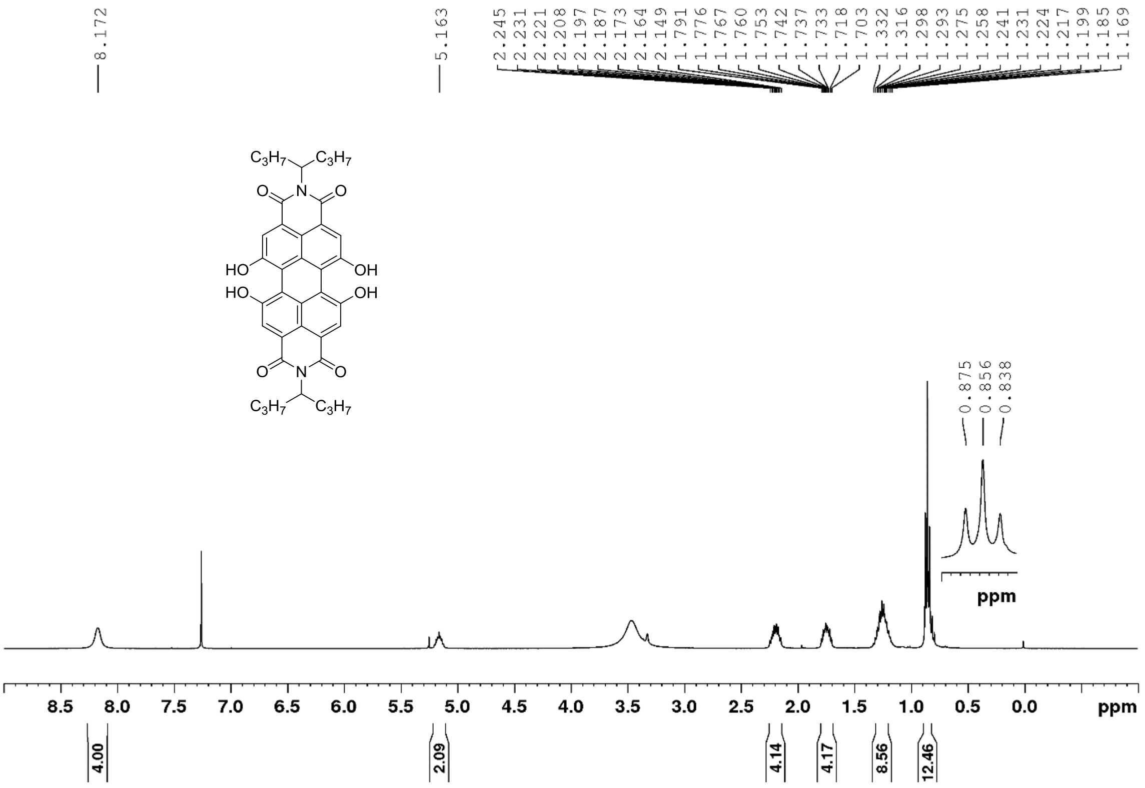

Figure S3. ^{1}H NMR of compound **HO-PBI-iC7** (400 MHz, $CDCl_3/CD_3OD$ 10:1, 298 K).

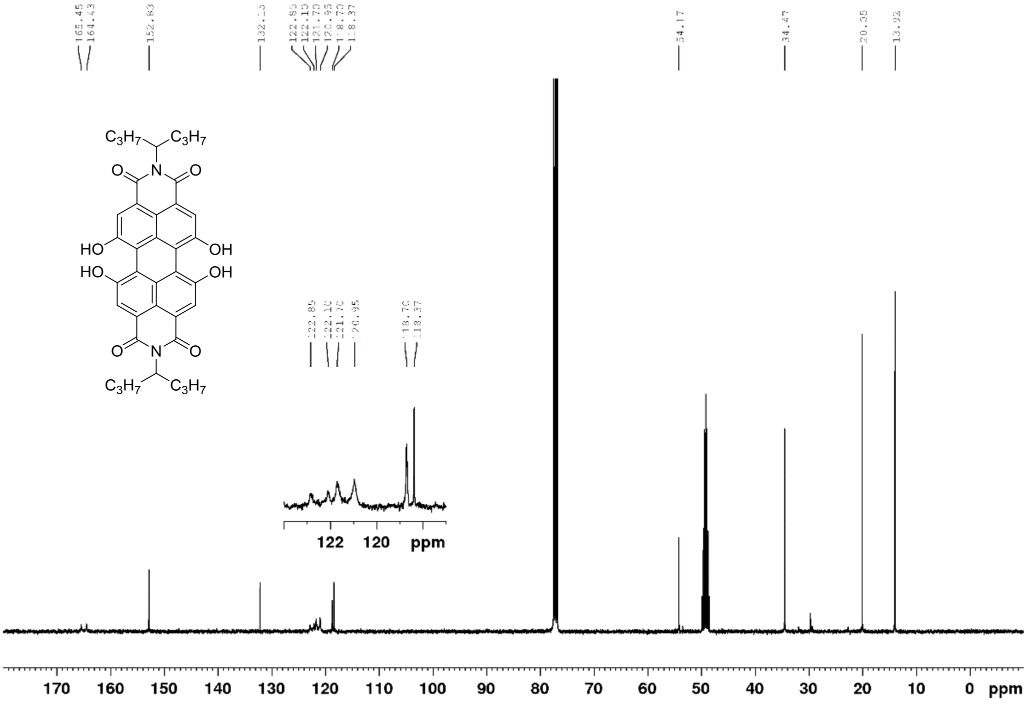

Figure S4. ^{13}C NMR of compound **HO-PBI-iC7** (101 MHz, $CDCl_3/CD_3OD$ 10:1, 298 K).

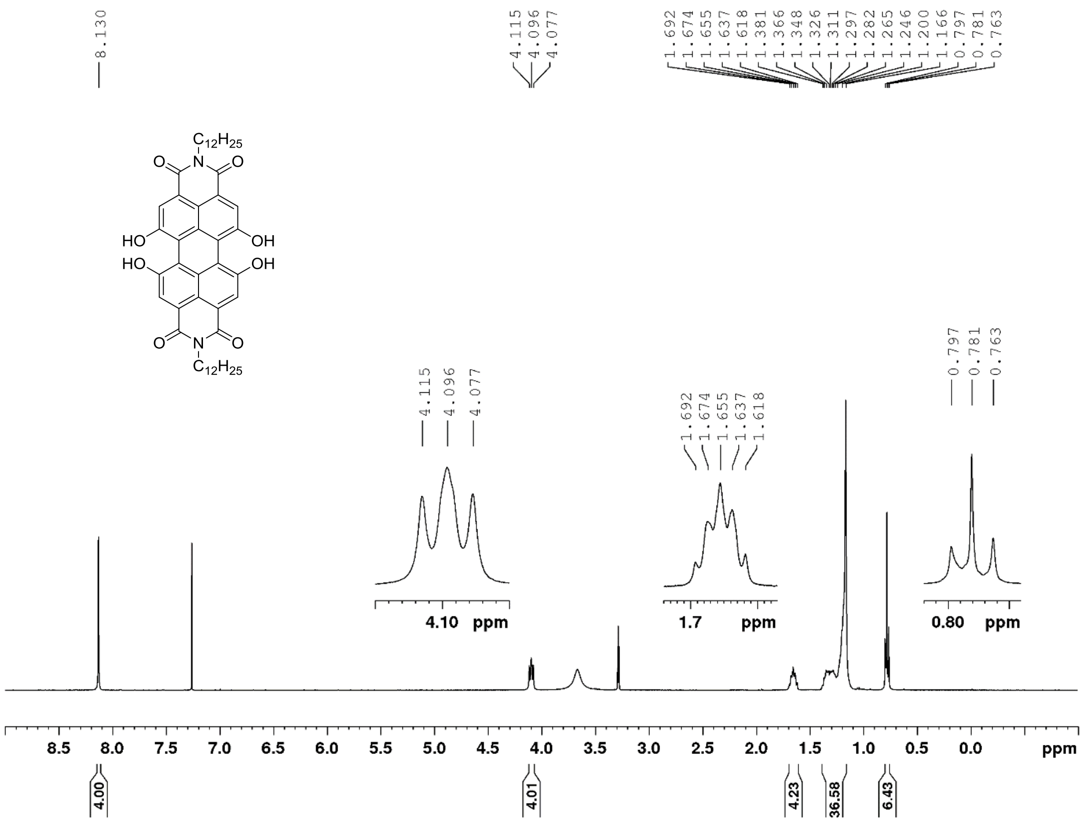

Figure S5. ^{1}H NMR of compound **HO-PBI-C12** (400 MHz, $CDCl_3/CD_3OD$ 10:1, 298 K).

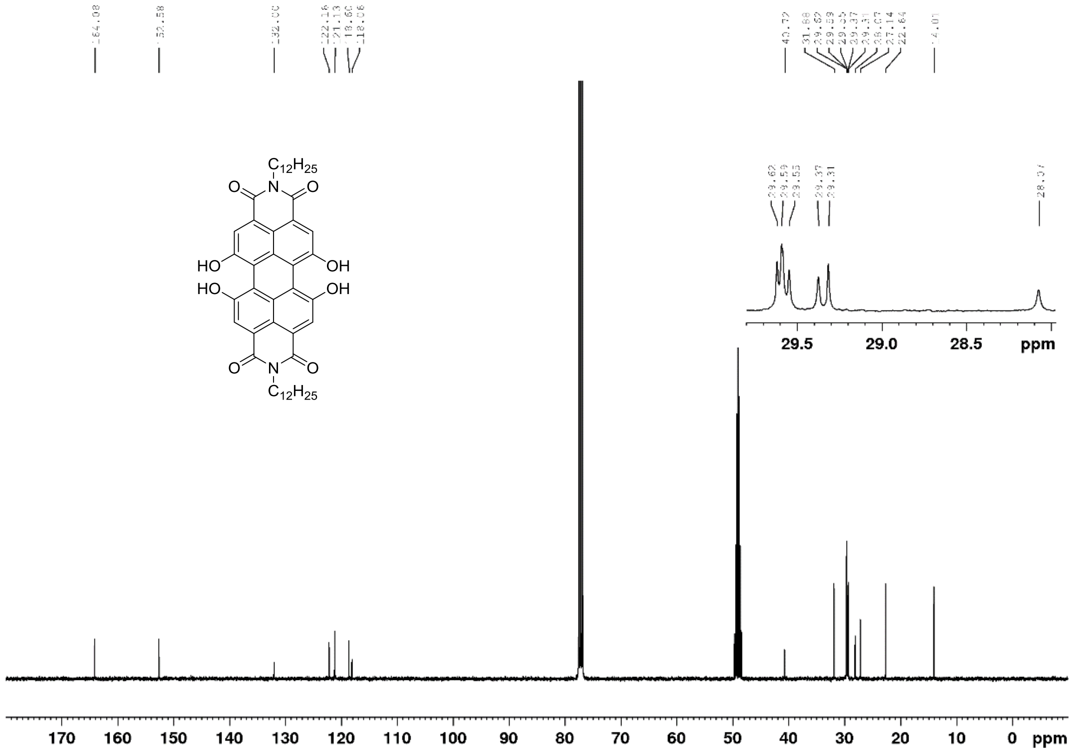

Figure S6. ^{13}C NMR of compound **HO-PBI-C12** (101 MHz, $CDCl_3/CD_3OD$ 10:1, 298 K).

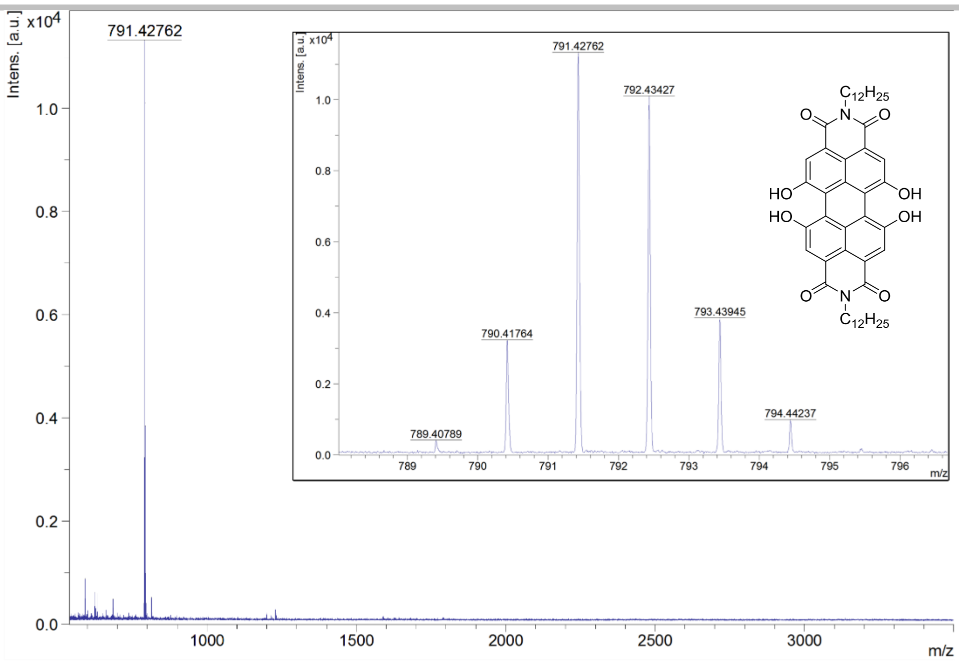

Acquisition Parameter

Spectrometer

positive voltage polarity POS
PIE delay 140 ns
Ion source voltage 1 20 kV
Ion source voltage 2 17.8 kV
Lens voltage 8 kV
Linear detector voltage 0 kV
Deflection on
Deflection mass

Reflector voltage 1 20.8 kV
Reflector voltage 2 10.9 kV
Reflector detector volt. 1.985 kV
Acquisition method name D:\Methods\flexControlMethods\Standard Methods\RP_100-2000_Da.par

Instrument Info

Instrument

Instrument type ultraflexTOF/TOF
Instrument serial 8276601.00641
Name of computer UTX-641
Operator ID or name FLEX-User
flexControl version flexControl 3.4.135.7
flexAnalysis version

Target

Target type
Target serial number
Position C17

Figure S7. HRMS (MALDI-TOF) spectrum of compound **HO-PBI-C12**.

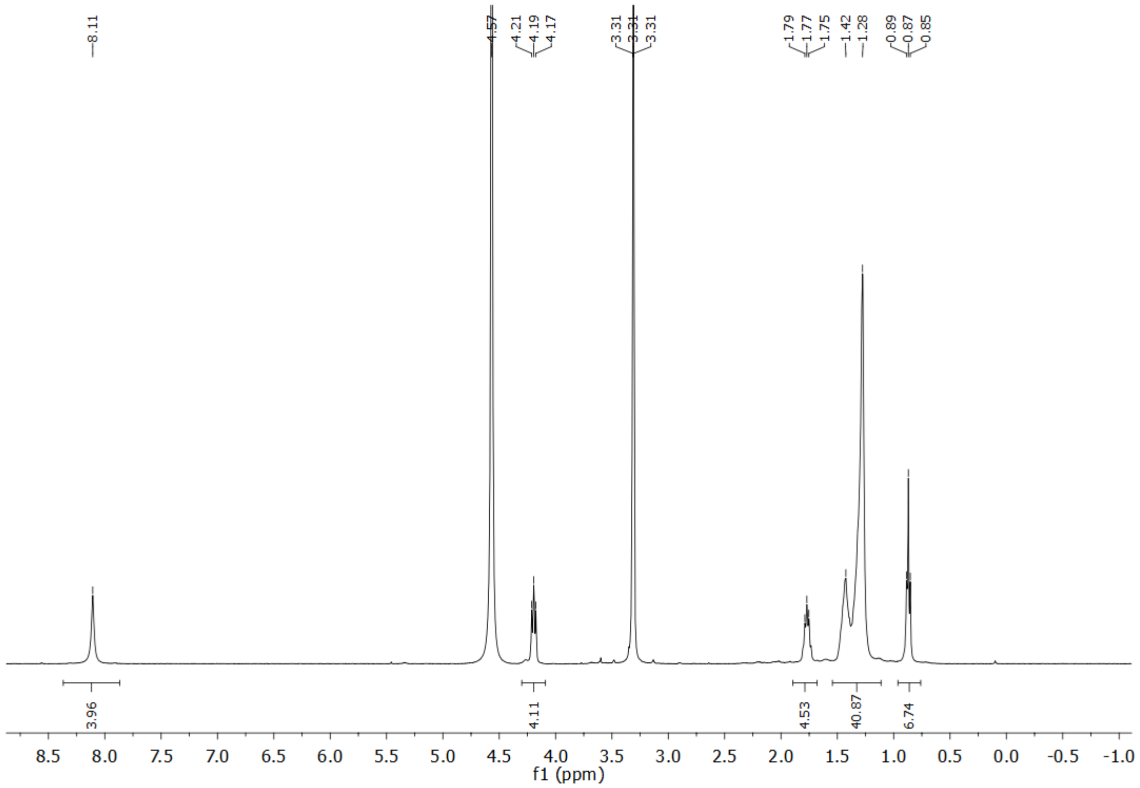

Figure S8. ^{1}H NMR of **the sodium salt of HO-PBI-C12** (400 MHz, CD_3OD, 326 K).

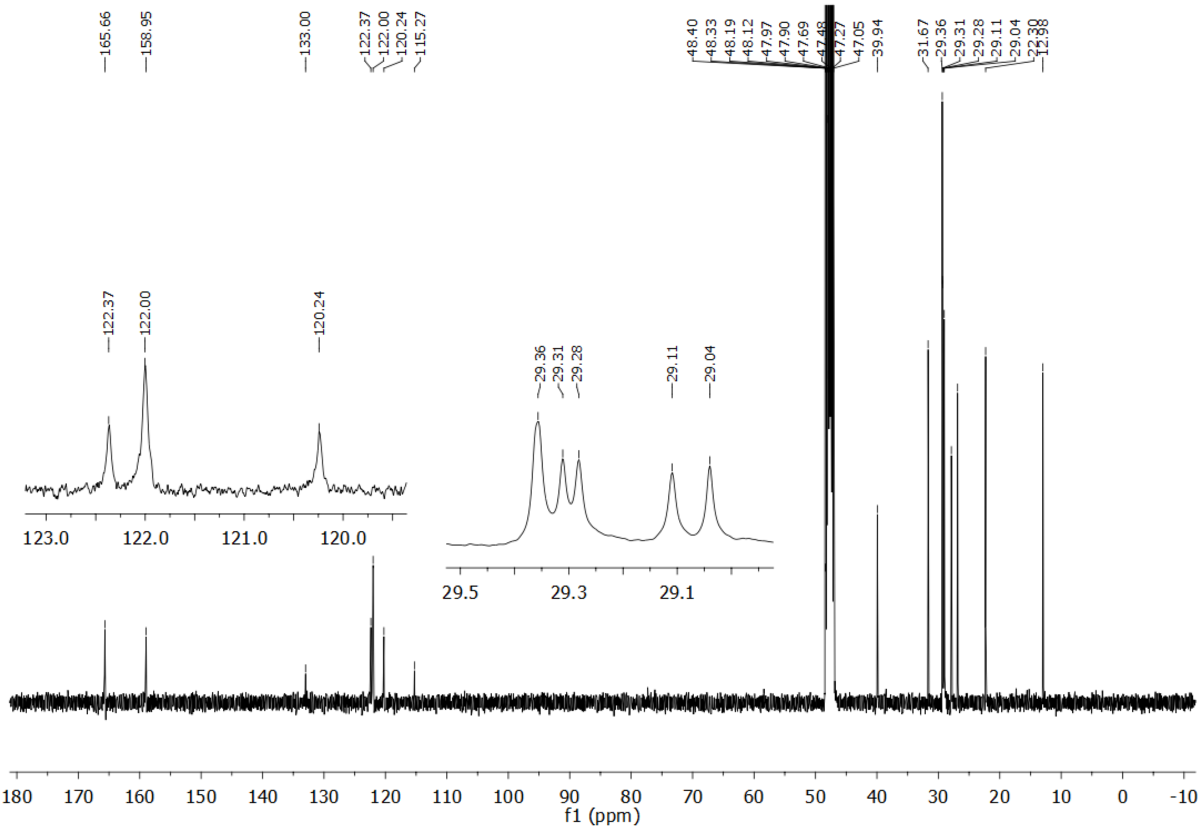

Figure S9. ^{13}C NMR of **the sodium salt of HO-PBI-C12** (101 MHz, CD_3OD, 326 K).

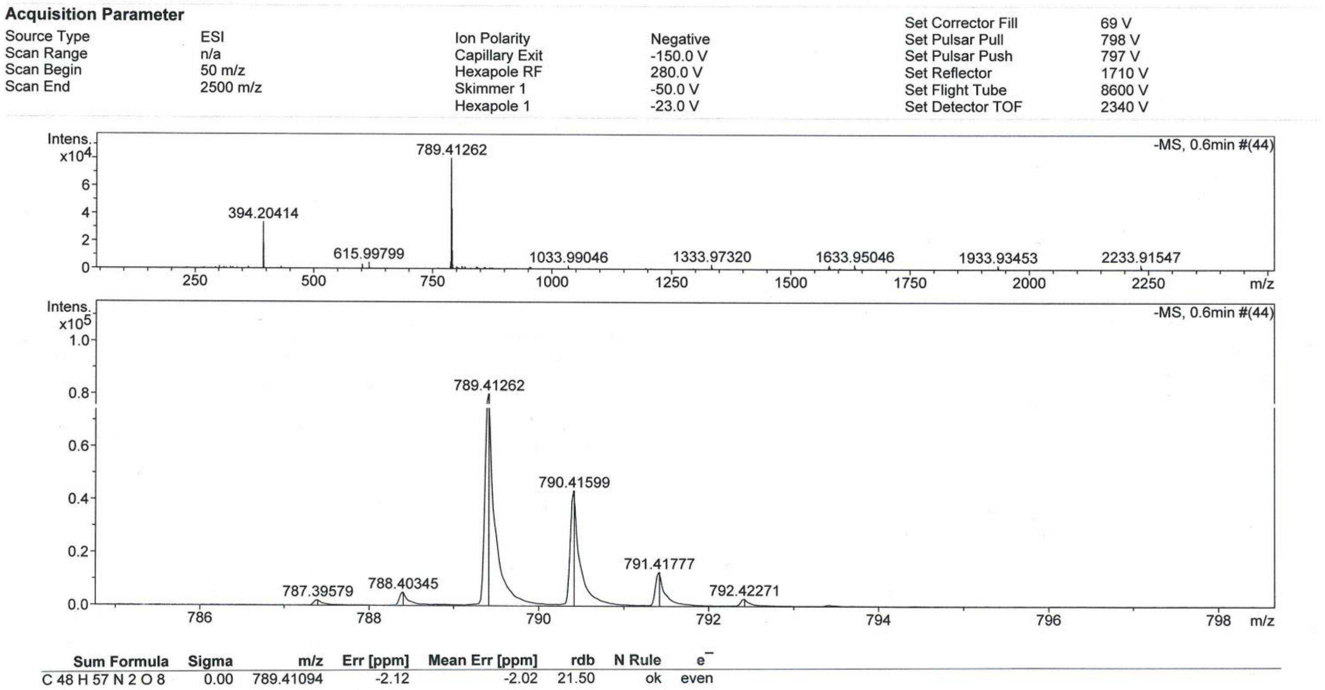

Figure S10. HRMS (ESI-TOF) spectrum of **the sodium salt of HO-PBI-C12**.

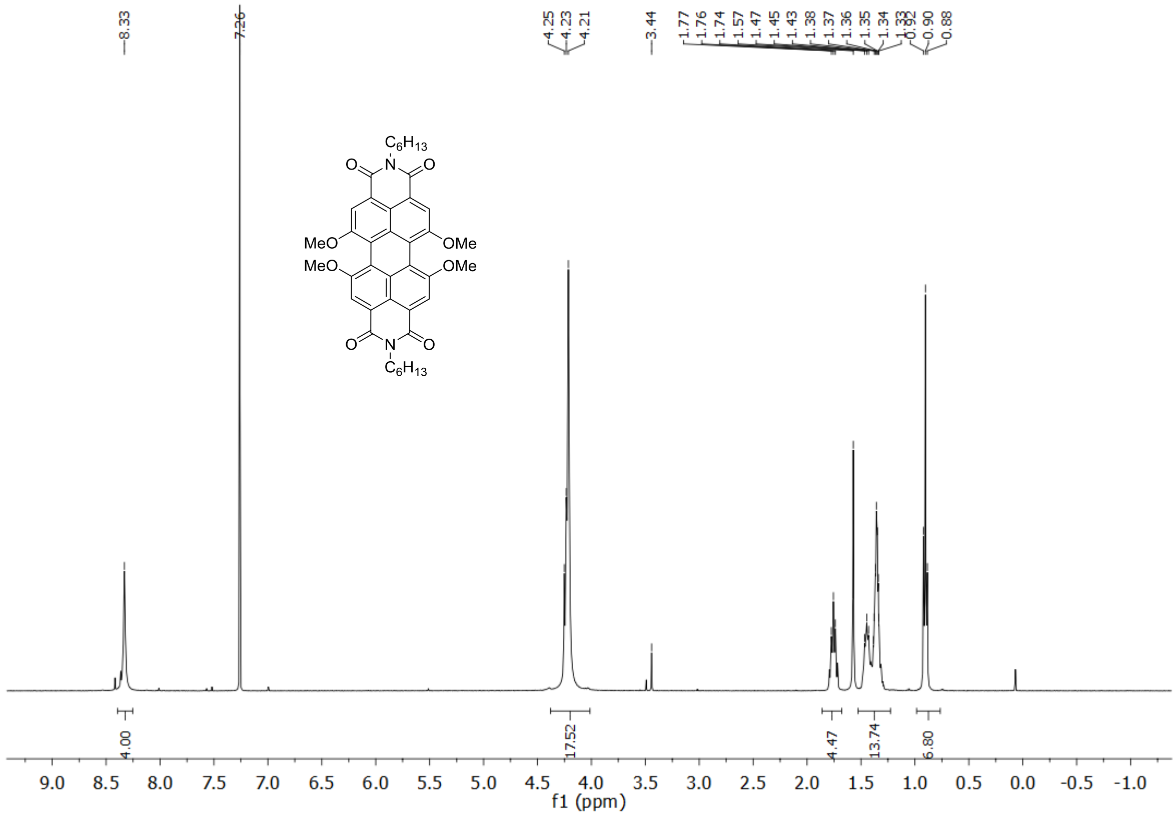

Figure S11. ^{1}H NMR of compound **MeO-PBI-C6** (400 MHz, $CDCl_3$, 298 K).

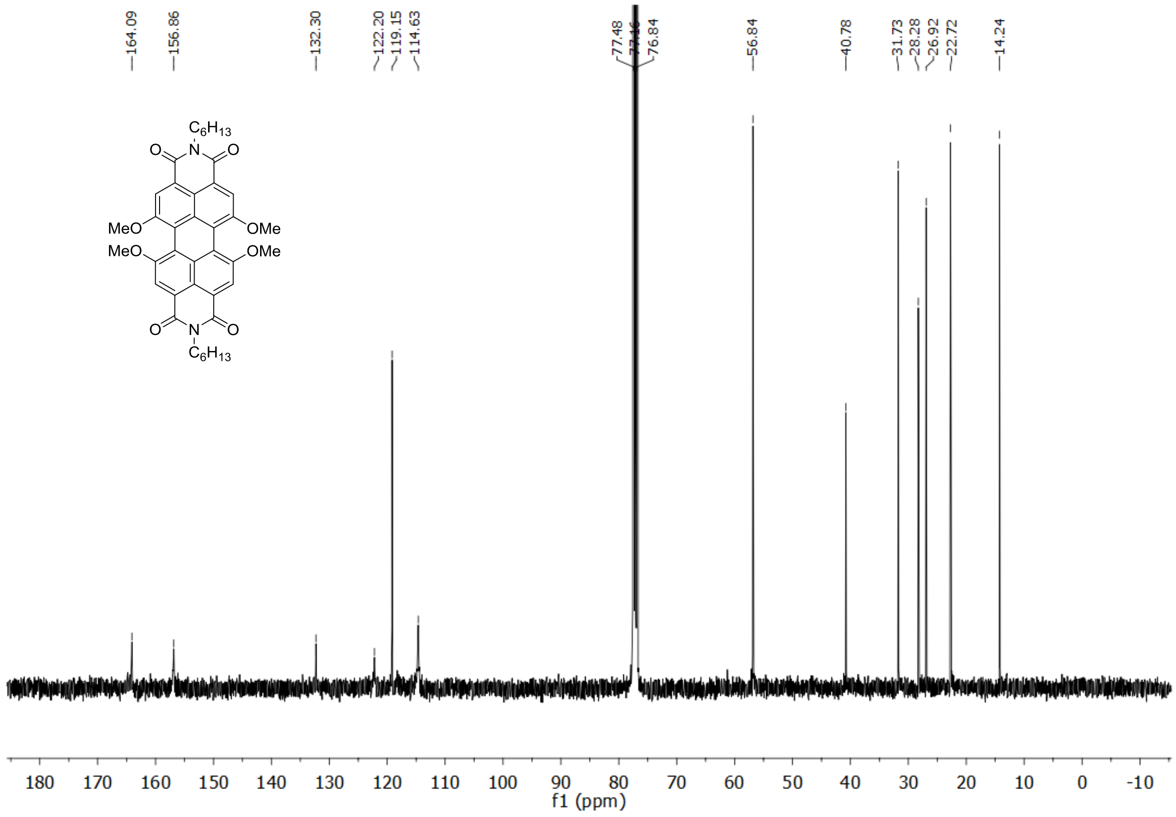

Figure S12. ^{13}C NMR of compound **MeO-PBI-C6** (101 MHz, $CDCl_3$, 298 K).

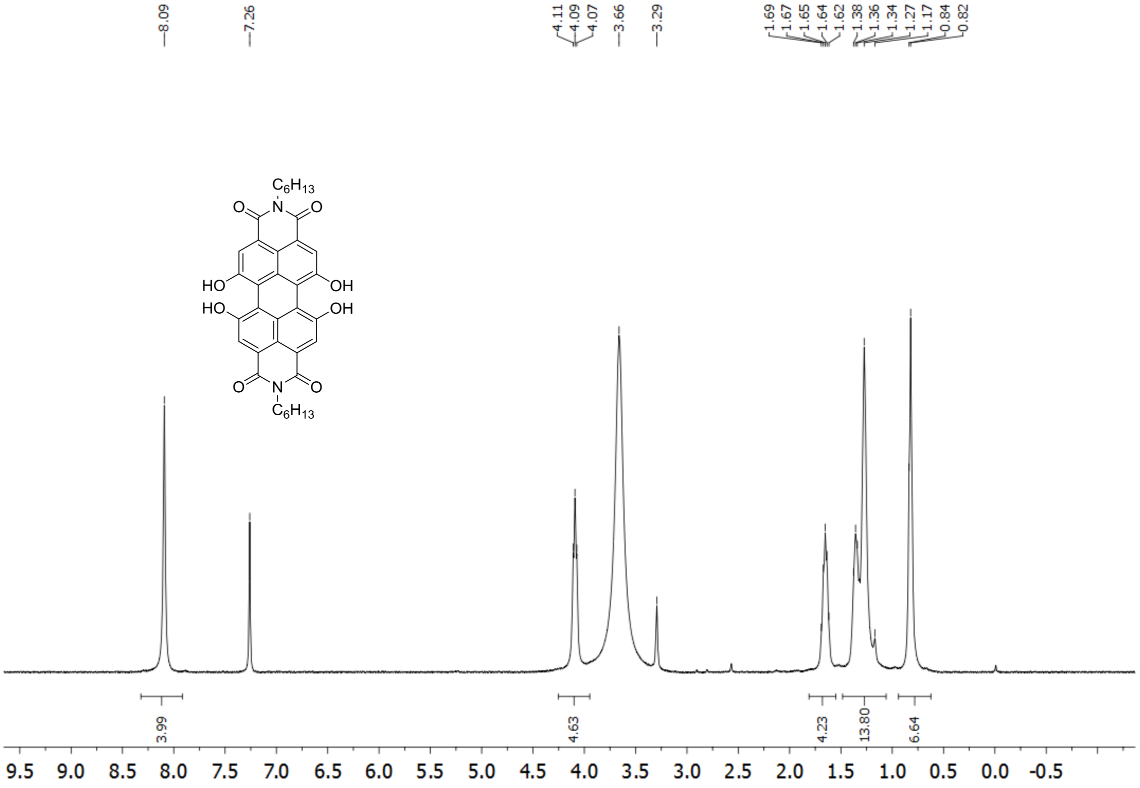

Figure S13. ^{1}H NMR of compound **HO-PBI-C6** (400 MHz, $CDCl_3/CD_3OD$ 10:1, 298 K).

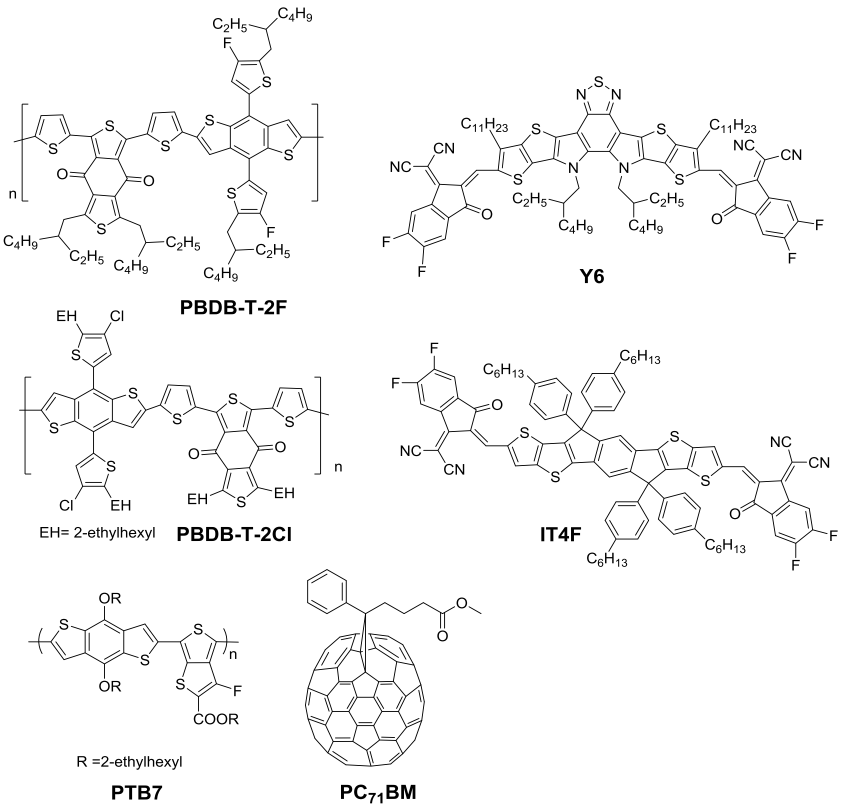

Figure S14. Non-fullerene and fullerene solar cell active layer materials utilized in the manuscript.

3. Solvent and pH-dependent absorption spectra of HO-PBI-C12

Absorption spectra of tetrahydroxy PBIs strongly depend on the polarity and nature of a solvent. For instance, **HO-PBI-C12** features a pink color in a chloroform solution, which corresponds to the absorption band at 556 nm (Figure 1). Titration with a base leads to the decrease in the intensity of the absorption band at 556 nm and emergence of a new broad band at 640 nm corresponding to the twofold deprotonated species (Figure 1 and Figure S15, green line), while the color of the solution changes from pink to cyan. Similar signatures can be observed in the spectra of a sodium salt of **HO-PBI-C12** in MeOH (Figure S15, red line) and even in spectra of **HO-PBI-C12** in CH_2Cl_2/MeOH (50:50), acetone/water (85:15), and THF/water (85:15) (Figure S15, blue, black, and orange lines, respectively) indicating that dianion is formed already upon addition of weakly basic co-solvents such as water or methanol.

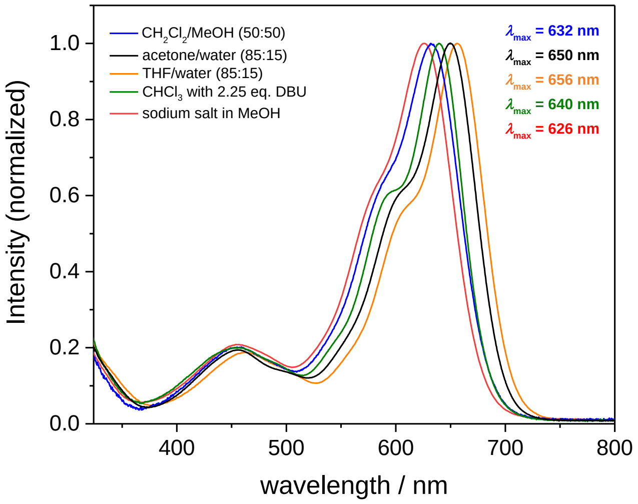

Figure. S15. Normalized UV-vis absorption spectra of **HO-PBI-C12** in CH_2Cl_2/MeOH 1:1 ($c = 7.3\cdot10^{-6}$ M, blue line), acetone/water 85:15 ($c = 2.9\cdot10^{-5}$ M, black line), THF/water 85:15 ($c = 2.2\cdot10^{-5}$ M, orange line), its deprotonated form in chloroform by addition of 2.25 eq. of DBU ($c = 1.9\cdot10^{-5}$ M green line), and its sodium salt in MeOH ($c = 3.1\cdot10^{-5}$ M, red line) at 298 K.

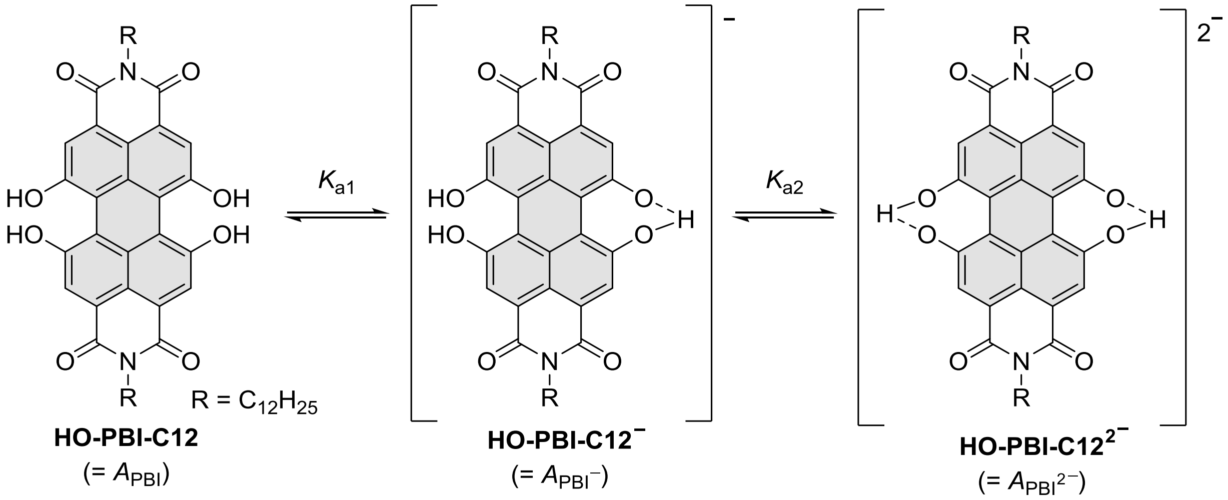

Scheme S1. Acid/base equilibrium of **HO-PBI-C12**. A_{PBI} denotes absorbance of a particular form of **HO-PBI-C12** (neutral form, monoanion and dianion).

The striking differences between absorption spectra of the neutral **HO-PBI-C12** and its ionized species facilitated spectrophotometric pK_a determination. Thus, **HO-PBI-C12** (0.11 mg, 1.44×10^{-7} mmol) was dissolved in a water-acetone mixture (5 mL, 15:85 (v/v)) and the sample was titrated with the aqueous solution of HCl.
The pH and absorption spectra of the solution were measured using a Mettler Toledo FiveGo pH meter FG2 equipped with a SI Analytics N 5800 BNC electrode and Jasco V-770 spectrophotometer with a 1.0-cm cell, respectively. The pH conditions from pH 6.3 to pH 3.4 were obtained by the titration of a **HO-PBI-C12** solution with 0.03 M HCl in steps of 2.5 µL and 0.06 M in steps of 5 µL (a total volume of 77.5 µL of HCl (aq.)). The pH conditions from pH 3.4 to pH 1.8 were obtained using 0.12 M and 1.20 M HCl in steps of 2.5 to 40 µL (a total volume of 182.5 µL of HCl (aq.)). The lowest pH value of 0.4 was achieved by the addition of 12 M aqueous HCl solution in steps from 5 to 30 µL (a total volume of 65 µL of HCl (aq.)). The pH and absorption spectrum of the resulting solution were measured at 25 °C after the addition of a certain amount of HCl (aq.).

Figure S16a displays the absorption spectra of **HO-PBI-C12** solution under various pH conditions. In the water/acetone (15:85) mixture, **HO-PBI-C12** showed a broad absorption spectrum with a maximum at 650 nm, which resembled the absorption spectrum of **HO-PBI-C12^{2-}** in chloroform produced by the addition of DBU (see Figure 1). Thus, water is sufficiently basic to promote deprotonation of **HO-PBI-C12**. A stepwise increase in the pH value entailed the gradual decrease in the intensity of the absorption band at 650 nm. These changes were accompanied by the increase in the intensity of the S_0-S_1 absorption band at 549 nm corresponding to the neutral species. The plot of absorbance at 650 nm vs pH value, along with the fitted curve, is shown in Figure S16b. The change of the absorbance proceeded in two steps which can be related to the twofold deprotonation (Scheme S1) by analogy to deprotonation with DBU.

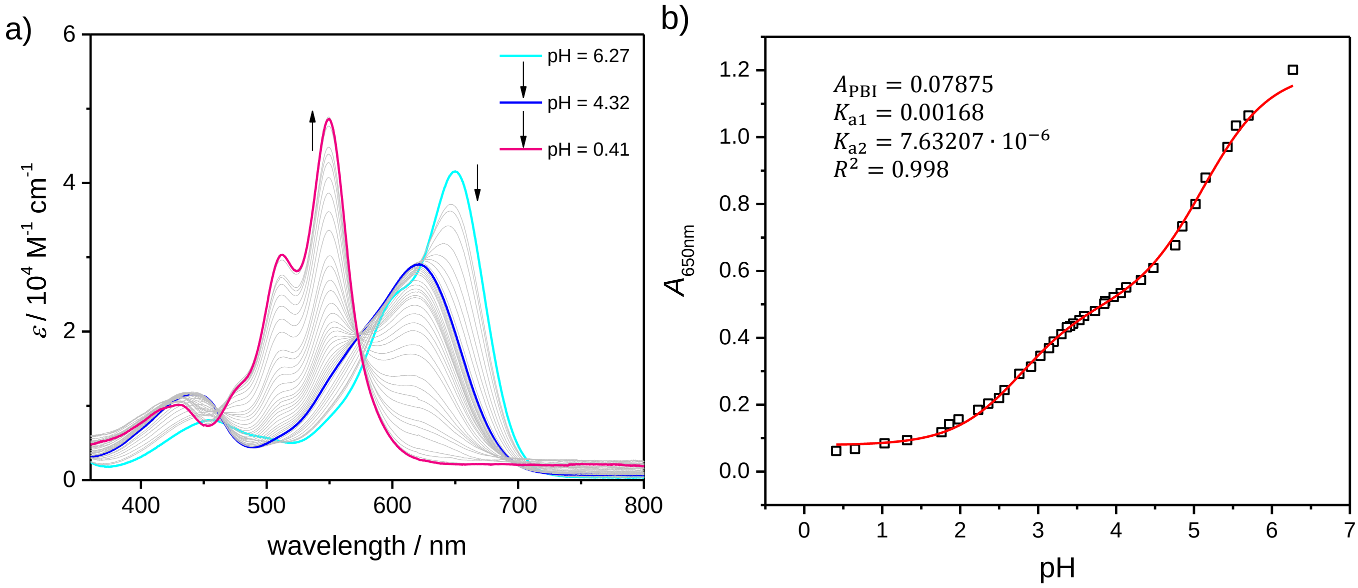

Figure S16. a) pH-dependent absorption spectra of **HO-PBI-C12** in acetone/water 85:15 (v/v) (c = 2.9·10^{-5} M) at 298 K. Arrows indicate changes upon addition of HCl (aq.). Absorption spectra of the initial solution of **HO-PBI-C12** at pH = 6.3 (cyan line), at pH = 4.3 (blue line) and at the end of the titration at pH = 0.4 (magenta line). b) Absorption changes at 650 nm *vs.* pH (scatter plot, black line) and fitting to the equation 1 (red solid line).

The pK_{a1} and pK_{a2} values for **HO-PBI-C12** were determined by the curve fitting according to the following equation:

$$A_{650\,nm} = \frac{A_{\mathrm{PBI}}+K_{\mathrm{a1}}\cdot 10^{\mathrm{pH}}\cdot A_{\mathrm{PBI}^{-}}+K_{\mathrm{a1}}\cdot K_{\mathrm{a2}}\cdot 10^{\mathrm{pH}}\cdot 10^{\mathrm{pH}}\cdot A_{\mathrm{PBI}^{2-}}}{1+K_{\mathrm{a1}}\cdot 10^{\mathrm{pH}}+K_{\mathrm{a1}}\cdot K_{\mathrm{a2}}\cdot 10^{\mathrm{pH}}\cdot 10^{\mathrm{pH}}} \qquad \text{(eq. 1)}^{[S4]}$$

where K_{a1} and K_{a2} are acid dissociation constants for each proton dissociation of **HO-PBI-C12**. A_{PBI}, $A_{\mathrm{PBI}^{-}}$, and $A_{\mathrm{PBI}^{2-}}$ denote absorbances of **HO-PBI-C12**, **HO-PBI-C12^{-}**, **HO-PBI-C12^{2-}**, respectively. The plateau in the plot $A_{635\,nm}$ vs. pH was used to derive the absorbance values $A_{\mathrm{PBI}^{-}}$, and $A_{\mathrm{PBI}^{2-}}$ of 0.51 and 1.20, respectively. The calculated pK_a values are pK_{a1} = 2.8 and pK_{a2} = 5.1 and indicate high acidity of tetrahydroxy PBI. The pK_{a2} value is higher than pK_{a1} as the second deprotonation is less feasible for the charged molecule **HO-PBI-C12^{-}**.

4. Coordination of Tetrahydroxy-PBIs and Zn(II) Metal ions

To get insight into the formation of zinc complexes of **HO-PBI** ligands, spectrophotometric titration studies were carried out (Figure S17). Thus, the DCM/MeOH solution of the sodium salt of **HO-PBI-C12** was titrated with the solution of $Zn(OTf)_2$ in the same solvent mixture. To ensure the constant concentration of PBI, a stock solution of PBI was prepared. Each time a portion of this solution was mixed with $Zn(OTf)_2$ prior to the addition of the titrant to the titrated PBI solution. The complexation progress was monitored by UV-vis absorption spectroscopy. During the titration a hypsochromic shift of the absorption maximum was observed, corresponding to a color change from cyan to dark blue. Accordingly, the initial absorption maximum at 630 nm decreased and the maximum was shifted to 621 nm. The hypsochromic shift was accompanied by the substantial deviation of the baseline from zero in the red end of the spectrum. The latter change and the hypsochromic shift of λ_{max} could originate from the presence of larger (colloidal) PBI^{n-}-Zn^{2+} metallosupramolecular species (charge depending on the deprotonation state of the ligand). Further addition of $Zn(OTf)_2$ (3.3 eq.) resulted in a hyperchromic shift of the spectrum, while the baseline in the red end of the spectrum approached again a zero value. Thus, the titration studies of **HO-PBI-C12** with the solution of $Zn(OTf)_2$ reveal two consecutive processes: initial formation of a PBI^{n-}-Zn^{2+} metallosupramolecular species species, followed by their degradation into smaller monomeric or oligomeric species at a higher Zn^{2+} concentration, i.e. coordination of excess amounts of Zn^{2+} acts as a terminating unit of the metallosupramolecular chain.

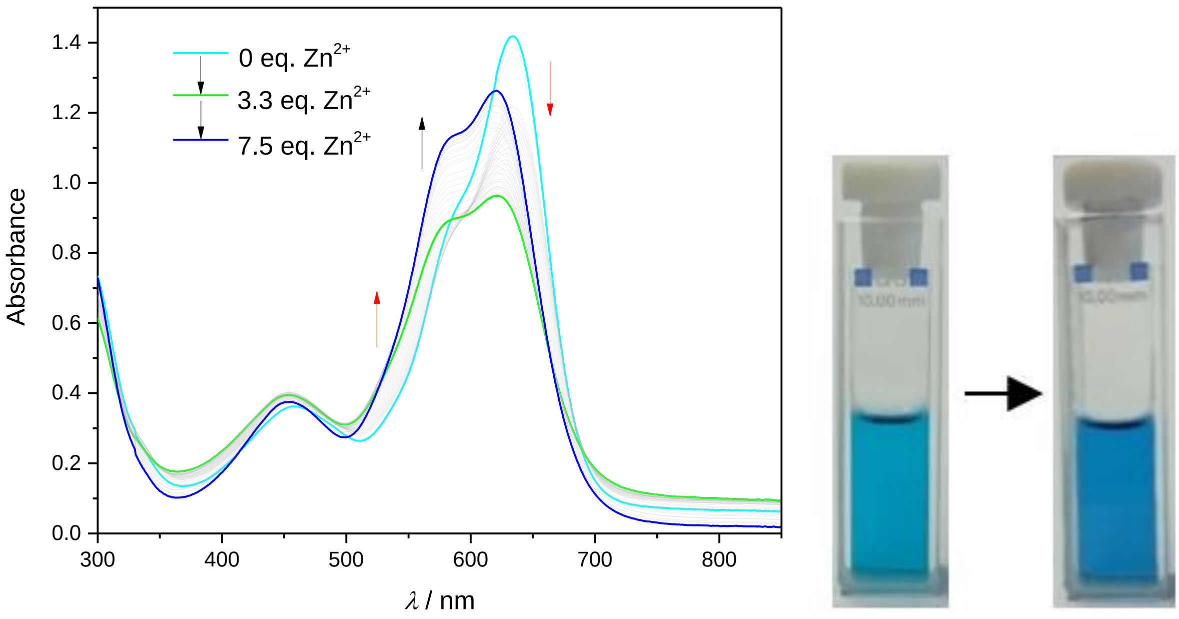

Figure S17. UV-vis-spectra of the titration of **HO-PBI-C12** (c = 4.1·10^{-5} M) with $Zn(OTf)_2$ in DCM/MeOH 1/1 and color changes in the cuvettes.

5. Electrochemistry

Electrochemical analysis allowed to assess the highest-occupied molecular orbital (HOMO) energy level of a sodium salt of **HO-PBI-C12** assuming the energy level of Fc/Fc^+ to be at −5.15 eV vs. vacuum[S5] (equation 2). The lowest occupied molecular orbital (LUMO) was determined based on the HOMO level and the optical band gap according to equation 3. The optical band gap was calculated according to equation 4.

$$E_{HOMO} = -e\,E_{1/2}^{ox} - 5.15 \text{ eV} \qquad \text{(eq. 2)}$$
$$E_{LUMO} = E_{HOMO} + (hc/\lambda_{max}) \qquad \text{(eq. 3)}$$
$$E_{gap} = E_{HOMO} - E_{LUMO} = hc/\lambda_{max} \qquad \text{(eq. 4)}$$

Redox potentials of the compound were measured by cyclic voltammetry (CV) in MeOH in the presence of Bu_4NPF_6 as a supporting electrolyte and calibrated versus ferrocenium/ferrocene (Fc^+/Fc). The cyclic voltrammogram is shown in Figure S18.

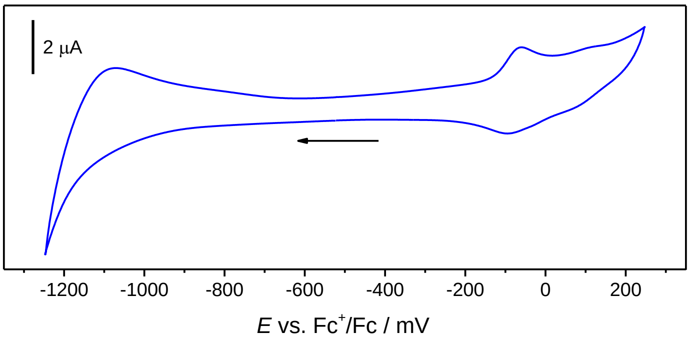

Figure S18. Cyclic voltammogram of a sodium salt of **HO-PBI-C12** (c ~ 2.8·10−4 M) in MeOH solution of n-Bu_4NPF_6 (0.1 M) at a scan rate 100 mV s^{-1}. The measurements were calibrated with an internal standard (ferrocenium/ferrocene).

The calculated HOMO/LUMO levels are:

$$E_{HOMO} = -5.09 \text{ eV}$$
$$E_{LUMO} = -3.11 \text{ eV}$$

A sodium salt of **HO-PBI-C12** displays drastically different solubility to its neutral form. While neutral **HO-PBI-C12** is scarcely soluble in pure MeOH, its sodium salt shows satisfactory solubility in this solvent. The poor solubility of **HO-PBI-C12** in MeOH and any other solvent suitable for cyclic voltammetry did not allow the electrochemical studies for this molecules.

6. Single crystal X-ray analysis

Single crystal X-ray diffraction data for **MeO-PBI-C6** were collected at 100 K on a Bruker D8 Quest Kappa Diffractometer with a Photon100 CMOS detector and multi-layered mirror monochromated $Cu_K\alpha$ radiation. The structure was solved using direct methods, expanded with Fourier techniques and refined with the Shelx software package.[S6] All non-hydrogen atoms were refined anisotropically. Hydrogen atoms were included in the structure factor calculation on geometrically idealized positions.

Crystal data for **MeO-PBI-C6** ($C_{40}H_{42}N_2O_8$): *Mr* = 678.75, 0.302 x 0.155 x 0.093 mm^3, triclinic space group $P\bar{1}$, a = 8.6742(5) Å, α = 98.921(2)°, b = 12.9536(8) Å, β = 97.416(2)°, c = 15.4813(9) Å, γ = 103.666(2)°, V = 1644.75(17) Å^3, Z = 2, ρ(*calcd.*) = 1.371 g·cm^{-3}, μ = 0.778 mm^{-1}, $F_{(000)}$ = 720, $GooF(F^2)$ = 1.038, R_1 = 0.0331, wR^2 = 0.0893 for I>2σ(I), R_1 = 0.0356, wR^2 = 0.0917 for all data, 6332 unique reflections [θ ≤ 72.342°] with a completeness of 97.4% and 457 parameters, 0 restraints.

Crystallographic data for the crystal structure of **MeO-PBI-C6** have been deposited in the Cambridge Crystallographic Data Center with CCDC as supplementary publication no. CCDC 1923126. These data can be obtained free of charge from The Cambridge Crystallographic Data Centre *via* www.ccdc.cam.ac.uk/data_request/cif.

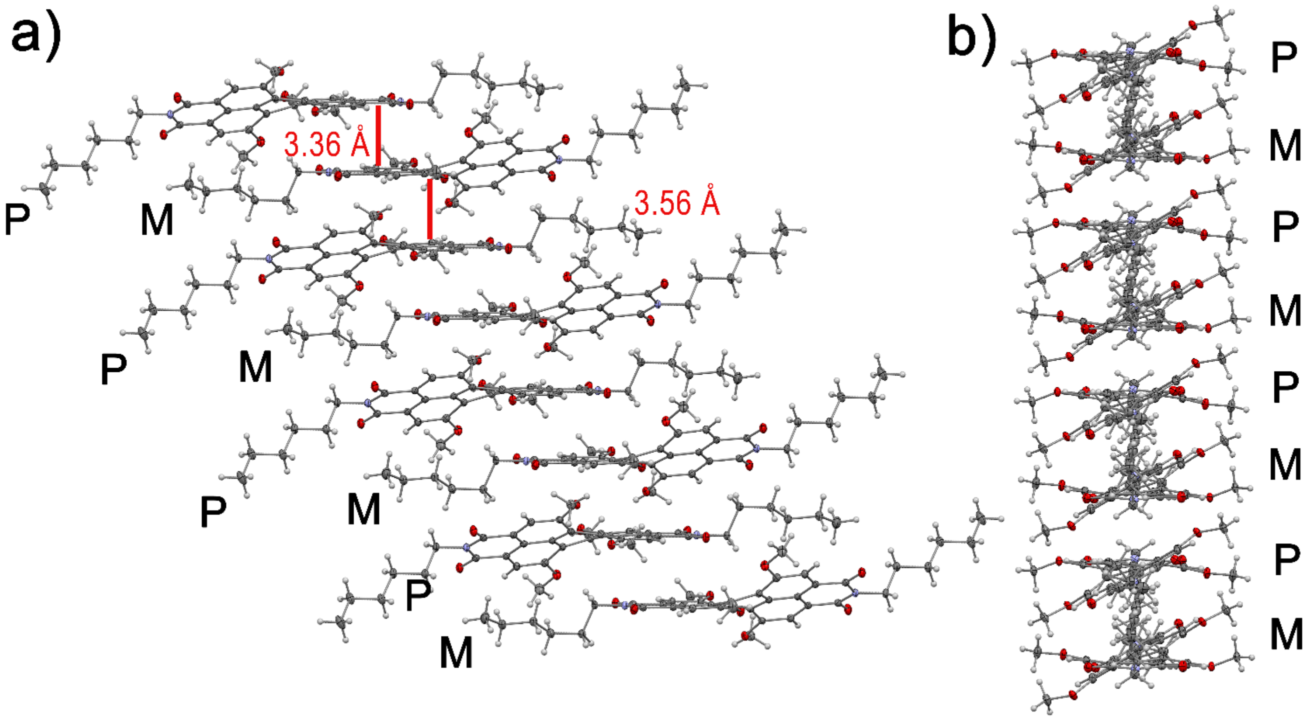

Figure S19. The stack of **MeO-PBI-C6**. b) The same stack viewed along the long axes of the PBI cores.

7. Fabrication and Characterization of OSCs

7.1 Fabrication and Characterization Method of Devices

Sol-gel derived pure and hybrid zinc oxide (ZnO) films were fabricated atop of indium tin oxide (ITO) or quartz substrates by spin-coating. Zinc acetate dihydrate was dissolved in the mixed solvent of 2-methoxyethanol and 2-aminoethanol (v/v 100:3) which composes the basic precursor solution. The PBI dye was directly added into the precursor solution in the specified ratio. Films were processed by spin-coating upon quartz or ITO and then immediately being annealed at 130 °C in 40 % relative humidity air for 1 hour. Then the substrates were transferred into a nitrogen glove box for spin-coating the active layer.

Patterned ITO-glass substrates were used as the cathode in the organic solar cells. Both substrates were cleaned by sonication in acetone, deionized water, detergent and isopropyl alcohol and dried in oven. The sol-gel derived ZnO and ZnO:**HO-PBI** interlayer (30 nm) were prepared according to the previously described procedure.[S7] The thickness of the films was determined by the surface profile (Bruker). The substrates were then transferred into a nitrogen glove box. The active layer was prepared by spin coating. A 10 nm MoO_3 layer and a 100 nm aluminum layer were thermally evaporated with a shadow mask to define the active area of devices (4.35 mm^2) and form the top anode. The PCE was determined from *J*–*V* curve measurements (using a Keithley 2400 source meter) under a 1 sun, AM 1.5G spectrum from a solar simulator (SAN-EI; 1000 W m^{-2}). Masks made using laser beam cutting technology to have a well-defined area of 435 mm^2 were attached to define the effective area for accurate measurement. All the masked and unmasked tests gave consistent results with relative errors within 5%. The solar simulator illumination intensity was determined using a monocrystal silicon reference cell calibrated by the National Renewable Energy Laboratory (NREL) and Konica Minolta Inc. (follows

JIS C8904-2). Theoretical J_{sc} values obtained by integrating the product of the EQE with the AM 1.5G solar spectrum agreed with the measured value to within 5%.

Electron-only devices were fabricated to measure the electron mobility using the space charge limited current (SCLC) method. The device structures are ITO/ Al / ZnO or ZnO:**HO-PBI** (90nm)/ Ca / Al to test the electron mobility of the device. The mobility was determined by fitting the dark current to the model of a single carrier SCLC, which is described by the equation of J_{sc}=(9/8) $\varepsilon_0\varepsilon_r\mu((V^2)/(L^3))$, where J_{sc} is the current density, μ is the zero-field mobility, ε_0 is the permittivity of free space, ε_r is the relative permittivity of the material, L is the thickness of the active layer, and V is the effective voltage. The effective voltage can be obtained by subtracting the built-in voltage (V_{bi}) and the voltage drop (V_s) from the substrate's series resistance from the applied voltage (V_{appl}), $V = V_{appl}-V_{bi}-V_s$. Here, the built-in voltage (V_{bi}) was estimated to be 0 V from the experimental dark current density-voltage characteristics. The V_s was regarded as 0 V since the series resistance of Al (~100 nm) on ITO was negligible. The electron-mobility can be calculated from the slope of the $J^{1/2}$~V curves in Child's region.

7.2 Photovoltaic Performance of OSCs with Various Cathode Interlayers and BHJ Layers

Table S1. Photovoltaic performance of devices based on 0%, 0.5%, 1%, 2% doping ratio ZnO:**HO-PBI-C12** interlayer using an inverted device structure of ITO/interlayer/active layer/MoO_3/Al.

Doping ratio	Active layer	V_{oc} [V]	J_{sc} [mA/cm^2]	*FF* [%]	PCE [%][a]
0 %	PTB7:PC$_{71}$BM	0.74	15.44	67.15	7.78
	PBDB-T-2Cl:IT4F	0.850	19.53	73.98	12.28
0.5 %	PTB7:PC$_{71}$BM	0.75	16.20	72.28	8.90
1 %	PTB7:PC$_{71}$BM	0.75	16.61	72.57	9.03
	PBDB-T-2Cl:IT4F	0.852	20.25	75.36	13.00
2 %	PTB7:PC$_{71}$BM	0.75	16.30	72.20	8.82
	PBDB-T-2Cl:IT4F	0.877	19.23	76.57	12.91

[a] Average data from over 10 devices.

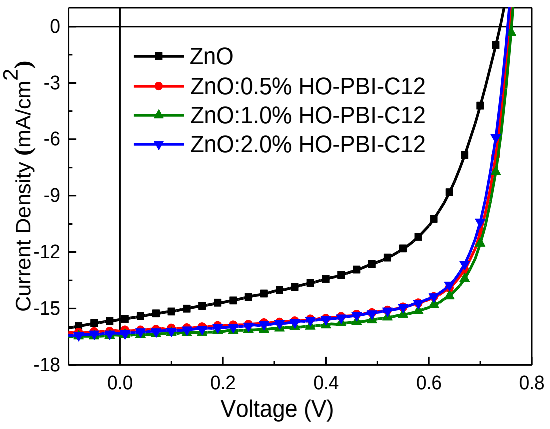

Figure S20. *J-V* characteristic for PTB7:PC$_{71}$BM active layer devices using 0 %, 0.5 %, 1 %, 2 % doping ratio.

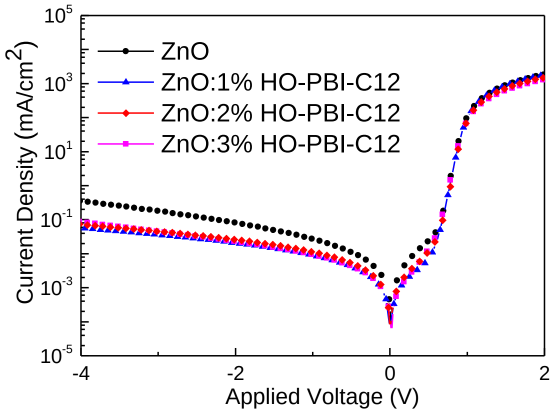

Figure S21. Dark condition *J-V* characteristic for inverted devices based on PBDB-T-2Cl:IT4F active layer using cathode interlayer with different doping ratio (0 %, 1 %, 2 %, 3 %). ZnO: **HO-PBI** devices show lower current in the reverse bias region, which indicates a higher rectification ratio and may due to the hole blocking properties of n-type molecule.

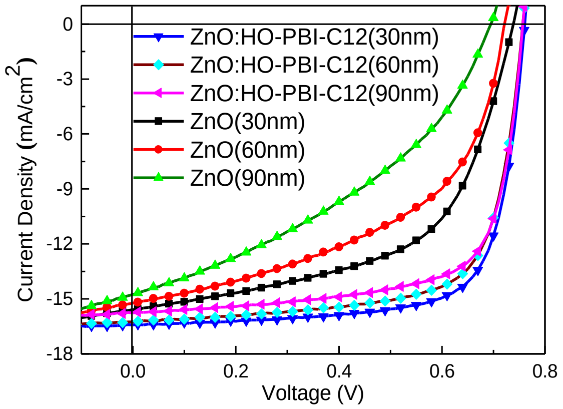

Figure S22. *J-V* characteristic for inverted devices based on PTB7:$PC_{71}BM$ active layer using cathode interlayer with thickness 30 nm, 60 nm, 90 nm.

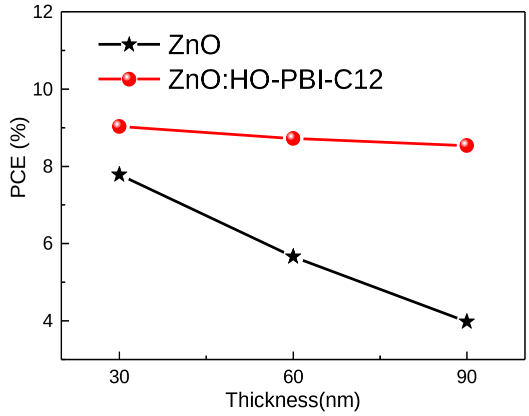

Figure S23. Power conversion efficiency-interlayer thickness dependence. Inverted devices based on PTB7:$PC_{71}BM$ active layer. The results show that **HO-PBI** doped interlayer films can eliminate the thickness effect and even work well at a high thickness of ~90 nm.

Table S2. Photovoltaic performance of devices based on interlayer thickness of 30 nm, 60 nm, and 90 nm using an inverted device structure of ITO/interlayer/PTB7:$PC_{71}BM$/MoO_3/Al.

Cathode interlayer	V_{oc} [V]	J_{sc} [mA/cm^2]	*FF* [%]	PCE [%][a]
ZnO (30 nm)	0.74	15.44	67.15	7.78 ± 0.42
ZnO (60 nm)	0.73	14.60	53.11	5.66 ± 0.89
ZnO (90 nm)	0.69	13.56	40.88	3.98 ± 1.33
ZnO:**HO-PBI-C12** (30 nm)	0.75	16.61	72.57	9.03 ± 0.25
ZnO: **HO-PBI-C12** (60 nm)	0.75	16.25	70.63	8.72 ± 0.41
ZnO: **HO-PBI-C12** (90 nm)	0.75	15.78	70.55	8.54 ± 0.96

[a] Average data from over 10 devices.

Table S3. Photovoltaic performance of devices after cathode interlayer annealing at 130 °C and 200 °C using an inverted device structure of ITO/interlayer/PBDB-T-2Cl:IT4F/MoO_3/Al.

Cathode interlayer	Annealing Temperature [° C]	V_{oc} [V]	J_{sc} [mA/cm^2]	*FF* [%]	PCE [%][a]
ZnO	130	0.851	18.65	75.90	12.04 ± 0.38 (12.28)
ZnO	200	0.843	19.46	74.33	12.19 ± 0.15 (12.33)
ZnO: **HO-PBI-C12**	130	0.852	20.18	75.74	12.99 ± 0.12 (13.17)
ZnO: **HO-PBI-C12**	200	0.866	19.16	76.13	12.63 ± 0.40 (12.91)

[a] Average data from over 15 devices. Best data in the bracket.

Table S4. Photovoltaic performance of devices based on 130 °C annealed ZnO interlayer using an inverted device structure of ITO/ZnO/active layer/MoO_3/Al.

Cathode Interlayer	Active layer	V_{oc} [V]	J_{sc} [mA/cm^2]	*FF* [%]	PCE [%][a]
ZnO	PBDB-T-2Cl:IT4F	0.851	18.65	75.90	12.04(12.28)
ZnO	PBDB-T-2Cl:Y6	0.830	24.60	72.73	14.87(15.20)
ZnO	PTB7:$PC_{71}BM$	0.74	15.44	67.15	7.78(8.07)

[a] Average data from over 10 devices. Best data in the brackets.

7.3 Electron Transporting Properties of the Cathode Interlayers

To elucidate the charge carrier mobilities of the interlayer, we have determined electron mobilities of ZnO:**HO-PBI** film by space charge limited current (SCLC) method.[S8] We fabricated electron-only devices with pristine and **HO-PBI-C12** doped ZnO. These results demonstrate that the electron mobility of pure ZnO can be improved by PBI doping from 5.01×10^{-4} cm^2 V^{-1} s^{-1} to 2.3×10^{-3} cm^2 V^{-1} s^{-1} (Figure S24 and Table S5), which may be attributed to the trap defect elimination by complexation. The decreased fluorescence intensity of ZnO:**HO-PBI-iC7** relative to that of ZnO support the reduced defect density in the doped system. In addition, such ZnO:**HO-PBI** film shows obvious photoconductive properties as illustrated in Figure S25 and S26. Defects in ZnO layer limit the electron transporting performance and we propose that photo-induced electron transfer from PBI molecules to ZnO can also eliminate the lattice defects and facilitate charge transport.[S7,S9]

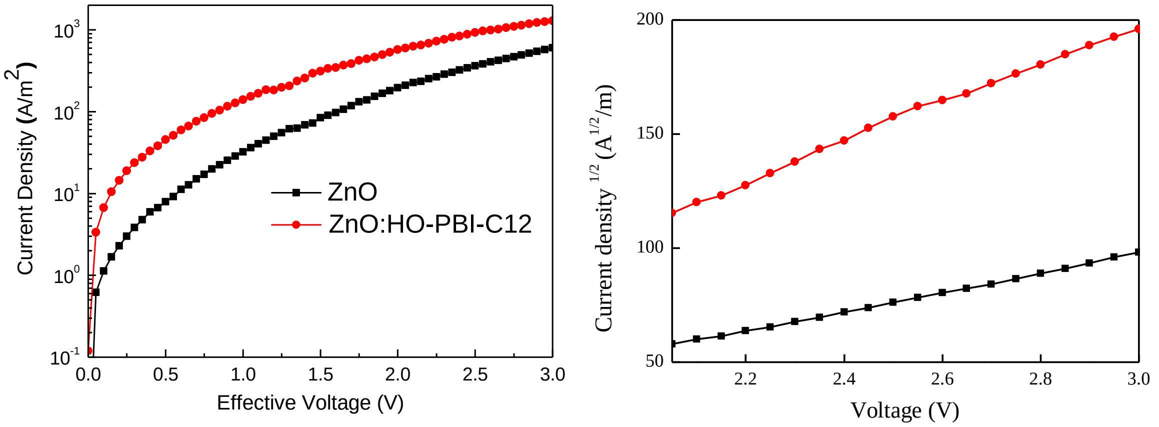

Figure S24. *J-V* curves (left) and Child region curves (right) of the electron-only devices for ITO/Al/interlayer (90 nm)/Ca/Al, in which the interlayer indicates ZnO or ZnO:**HO-PBI-C12**.

Table S5. Electron mobility based on fitting of Mott-Gurney law.

Electron-only device	Electron Mobility μ_e [cm^2 V^{-1} s^{-1}]
ITO/Al/ZnO/Ca/Al	5.01×10^{-4}
ITO/Al/ZnO:**HO-PBI-C12**/Ca/Al	2.30×10^{-3}

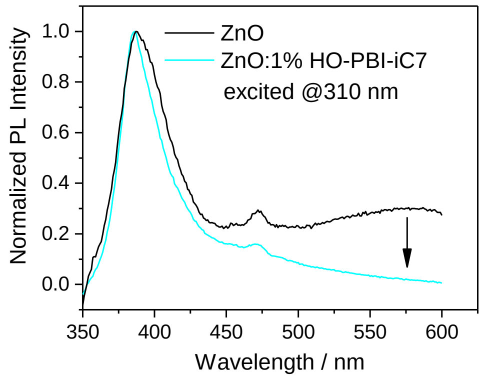

Figure S25. Photoluminescence spectra of pristine ZnO and **HO-PBI-iC7** embedded ZnO. The diffuse photoluminescence signal from 500 nm to 600 nm is caused by different trap-levels, which can be passivated by PBIs.

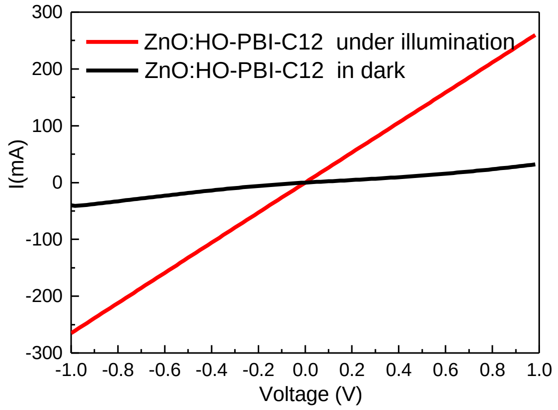

Figure S26. Current-voltage curves of ITO/ZnO:**HO-PBI-C12**(~90 nm)/Al from -1 V to 1 V. Higher conductivity can be observed after illumination due to photo-conductive behaviour.

7.4 Surface Morphology of Cathode Interlayer Thin Films

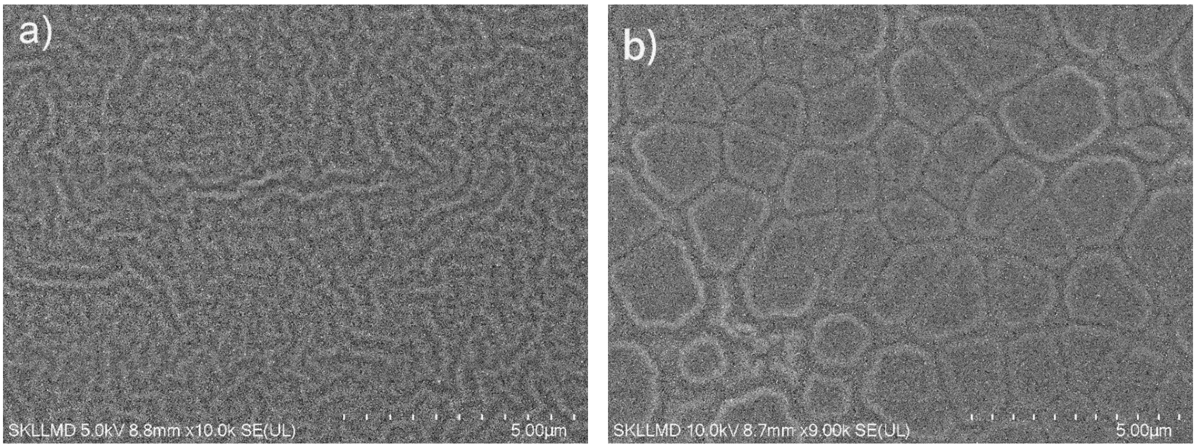

Figure S27. SEM images of pure ZnO (a) and ZnO:**HO-PBI-iC7** (b) on Silicon substrates, following the same processing method as devices.

References

[S1] P. Leowanawat, A. Nowak-Król, F. Würthner, *Org. Chem. Front.* **2016**, *3*, 537- 544.

[S2] A. Nowak-Król, M. I. S. Röhr, D. Schmidt, F. Würthner, *Angew. Chem. Int. Ed.* **2017**, *56*, 11774-11778; *Angew. Chem.* **2017**, *129*, 11936-11940.

[S3] A. J. Fry in *Laboratory Techniques in Electroanalytical Chemistry*, 2nd ed. (Eds.: P. T. Kissinger, W. R. Heineman), Marcel Dekker Ltd, New York, **1996**, p. 481.

[S4] X. Subirats, E. Fuguet, M. Rosés, E. Bosch, C. Ràfols, *Methods for pKa Determination (I): Potentiometry, Spectrophotometry, and Capillary Electrophoresis* in *Reference Module in Chemistry, Molecular Sciences and Chemical Engineering* (Ed.: J. Reedijk), Elsevier, Waltham, MA, **2015**.

[S5] a) N. G. Connelly, W. E. Geiger, *Chem. Rev.* **1996**, *96*, 877–910; b) W. N. Hansen, G. J. Hansen, *Phys. Rev. A* **1987**, *36*, 1396–1402; c) C. M. Cardona, W. Li, A. E. Kaifer, D. Stockdale, G. C. Bazan, *Adv. Mater.* **2011**, *23*, 2367–2371.

[S6] G. M. Sheldrick, *Acta Crystallogr.* A **2008**, *64*, 112-122.

[S7] L. Nian, W. Zhang, N. Zhu, L. Liu, Z. Xie, H. Wu, F. Würthner, Y. Ma, *J. Am. Chem. Soc.* **2015**, *137*, 6995-6998.

[S8] W. Chandra, L. K. Ang, K. L. Pey, C.M. Ng, *Appl. Phys. Lett.* **2007**, *90*, 152505.

[S9] T. Hu, F. Li, K. Yuan, Y. Chen, *ACS Appl. Mater. Interfaces* **2013**, *5*,5763-5770.
